# Supplementary material for: Evaluation of an intervention to provide brief support and personalized feedback on food shopping to reduce saturated fat intake (PC-SHOP): A randomized controlled trial
Source: PLoS Med. 2020 Nov 5;17(11):e1003385. doi: 10.1371/journal.pmed.1003385 (PMC7643942; doi:10.1371/journal.pmed.1003385)
Supplement: S1 Appendix — (DOCX) [file pmed.1003385.s001.docx]

**Study Title:**

Primary Care Shopping Intervention for Cardiovascular Disease Prevention

**Internal Reference Number / Short title:** PC-SHOP

**Ethics Ref:** 17/SC/0168

**Sponsor PID:** 12408

**ISRCTN:** 14279335

**Date and Version No:**

Version 4.0, 11^th^ December 2018

| **Chief Investigator:** | Dr. Carmen Piernas-Sanchez, Researcher,  Nuffield Department of Primary Care Health Sciences |
| --- | --- |
| **Investigators:** | Prof. Susan Jebb & Prof. Paul Aveyard,  Nuffield Department of Primary Care Health Sciences |
| **Sponsor:** | University of Oxford |
| **Funder:** | NIHR CLAHRC Oxford |
| **Chief Investigator Signature:** | 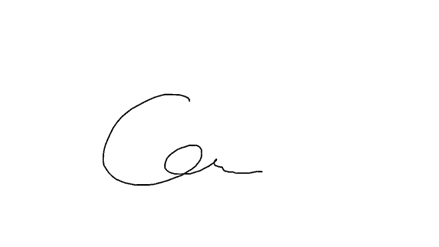  ………………………………………………………………………………………………… |

The investigators declare no potential conflicts of interest.

**Confidentiality Statement**

This document contains confidential information that must not be disclosed to anyone other than the Sponsor, the Investigator Team, host organisation, and members of the Research Ethics Committee, unless authorised to do so.

**TABLE OF CONTENTS**

[1. KEY CONTACTS 4](#_Toc279850)

[2. SYNOPSIS 6](#_Toc279851)

[3. ABBREVIATIONS 8](#_Toc279852)

[4. BACKGROUND AND RATIONALE 9](#_Toc279853)

[5. OBJECTIVES AND OUTCOME MEASURES 12](#_Toc279854)

[6. STUDY DESIGN 17](#_Toc279855)

[7. PARTICIPANT IDENTIFICATION 18](#_Toc279856)

[7.1. Study Participants 18](#_Toc279857)

[7.2. Inclusion Criteria 18](#_Toc279858)

[7.3. Exclusion Criteria 18](#_Toc279859)

[8. STUDY PROCEDURES 20](#_Toc279860)

[8.1. Screening and Recruitment 20](#_Toc279861)

[8.1. Eligibility Assessment 20](#_Toc279862)

[8.2. Informed Consent 20](#_Toc279863)

[8.3. Randomisation, blinding and code-breaking 21](#_Toc279864)

[8.4. Baseline Assessments 21](#_Toc279865)

[8.5. Subsequent Visits 22](#_Toc279866)

[8.5.1. Three-month follow up assessment 22](#_Toc279867)

[8.6. Food shopping data 23](#_Toc279868)

[8.7. Sample Handling 23](#_Toc279869)

[8.8. Alternative scheduling for follow-ups 23](#_Toc279870)

[8.9. Participant reimbursement 24](#_Toc279871)

[8.10. Discontinuation/Withdrawal of Participants from Study 24](#_Toc279872)

[8.11. Definition of End of Study 24](#_Toc279873)

[8.12. Qualitative sub-study 24](#_Toc279874)

[9. INTERVENTIONS 28](#_Toc279875)

[9.1. Intervention 1: Brief advice session 28](#_Toc279876)

[9.2. Intervention 2: Brief advice session and shopping report 29](#_Toc279877)

[9.3. Control group 29](#_Toc279878)

[10. SAFETY REPORTING 30](#_Toc279879)

[10.1. Definition of Serious Adverse Events 30](#_Toc279880)

[10.2. Reporting Procedures for Serious Adverse Events 30](#_Toc279881)

[11. STATISTICS AND ANALYSIS 31](#_Toc279882)

[11.1. Description of Statistical Methods 31](#_Toc279883)

[11.2. The Number of Participants 31](#_Toc279884)

[11.3. Analysis of Outcome Measures 32](#_Toc279885)

[12. DATA MANAGEMENT 33](#_Toc279886)

[12.1. Access to Data 33](#_Toc279887)

[12.2. Data Recording and Record Keeping 33](#_Toc279888)

[12.3. Study Management 33](#_Toc279889)

[13. QUALITY ASSURANCE PROCEDURES 35](#_Toc279890)

[13.1. Quality Control and Quality Assurance Procedures 35](#_Toc279891)

[13.2. Risk Assessment 35](#_Toc279892)

[13.3. Trial Steering Committee / Data Monitoring Committee 35](#_Toc279893)

[14. ETHICAL AND REGULATORY CONSIDERATIONS 36](#_Toc279894)

[14.1. Declaration of Helsinki 36](#_Toc279895)

[14.2. Guidelines for Good Clinical Practice 36](#_Toc279896)

[14.3. Approvals 36](#_Toc279897)

[14.4. Reporting 36](#_Toc279898)

[14.5. Participant Confidentiality 36](#_Toc279899)

[14.6. Expenses and Benefits 37](#_Toc279900)

[15. FINANCE AND INSURANCE 38](#_Toc279901)

[15.1. Funding 38](#_Toc279902)

[15.2. Insurance 38](#_Toc279903)

[16. PUBLICATION POLICY 39](#_Toc279904)

[17. REFERENCES 40](#_Toc279905)

[18. APPENDIX A: STUDY FLOW CHART 41](#_Toc279906)

[19. APPENDIX B: SCHEDULE OF STUDY PROCEDURES 42](#_Toc279907)

[20. APPENDIX C: QUALITATIVE SUB-STUDY TOPIC GUIDE 1](#_Toc279908)

[21. APPENDIX C: AMENDMENT HISTORY 5](#_Toc279909)

# KEY CONTACTS

| **Chief Investigator** | Dr. Carmen Piernas-Sanchez  Nuffield Department of Primary Care Health Sciences,  University of Oxford  Radcliffe Primary Care Building, Woodstock Road, Oxford, OX2 6GG  [carmen.piernas-sanchez@phc.ox.ac.uk](mailto:carmen.piernas-sanchez@phc.ox.ac.uk) |
| --- | --- |
| **Co-Investigators** | Professor Susan Jebb  Nuffield Department of Primary Care Health Sciences,  University of Oxford  Radcliffe Primary Care Building, Woodstock Road, Oxford, OX2 6GG  [susan.jebb@phc.ox.ac.uk](mailto:susan.jebb@phc.ox.ac.uk)    Professor Paul Aveyard  Nuffield Department of Primary Care Health Sciences,  University of Oxford  Radcliffe Primary Care Building, Woodstock Road, Oxford, OX2 6GG  [paul.aveyard@phc.ox.ac.uk](mailto:paul.aveyard@phc.ox.ac.uk) |
| **Clinical Trials Unit** | Claire Madigan  Senior Trial Manager  Primary Care Clinical Trials Unit  Nuffield Department of Primary Care Health Sciences,  University of Oxford  Radcliffe Primary Care Building, Woodstock Road, Oxford, OX2 6GG  [claire.madigan@phc.ox.ac.uk](mailto:claire.madigan@phc.ox.ac.uk) |
| **Sponsor** | University of Oxford  University Research Services  Joint Research Office  Block 60- Churchill Hospital  Oxford  OX3 7LE  [ctrg@admin.ox.ac.uk](mailto:ctrg@admin.ox.ac.uk) |
| **Funder** | The National Institute for Health Research (NIHR) Collaboration for Leadership in Applied Health Research and Care Oxford at Oxford Health NHS Foundation Trust  Dr Sara Ward: CLAHRC ManagerGrants P2-604; P2-604x; P2-611  [sara.ward@phc.ox.ac.uk](mailto:alex.gardiner@phc.ox.ac.uk)  The NIHR School of Primary Care Research (SPCR)  Grant 405 |
| **Trial Management Group** | Members:  Dr. Carmen Piernas-Sanchez  Prof. Paul Aveyard  Prof. Susan Jebb  Ms Charlotte Lee (Research Assistant)  Dr. Claire Madigan  Dr. Jason Oke (Trial Statistician) |

# SYNOPSIS

| **Study Title** | **P**rimary **C**are **Shop**ping Intervention for Cardiovascular Disease Prevention | |
| --- | --- | --- |
| **Internal ref. no. / short title** | PC-SHOP | |
| **Study Design** | Individually randomised, 3 arm parallel group study | |
| **Study Participants** | Adults ≥18 years of age with high LDL-cholesterol who are primarily responsible for household shopping and shop at Tesco at least once a week using Tesco’s storecard. | |
| **Planned Sample Size** | 112 total, 48 participants in each intervention group and 16 in the control group | |
| **Planned Study Period** | 24 months  Recruitment of practices will take 2 months, recruitment of participants will take around 10 months and each participant will be followed up for 3 months. The qualitative study will take approximately 9 months to complete. | |
|  | **Objectives** | **Outcome Measures** |
| **Primary** | To test a behavioural intervention aiming to promote a reduction in saturated fat (SFA) intake through brief advice from a qualified health professional alone or in combination with feedback on food purchasing behaviours against usual care. | Change in mean %SFA intake, measured using 2 x 24h dietary recalls twice (once at baseline and once 3 months later), using a validated web based questionnaire |
| **Secondary** | 1. Change in SFA content of food purchases  2. Biochemical markers of CVD risk  3. Change in food intake patterns | 1. 3-month changes in mean %SFA from total purchases; proportion of food items with low SFA (e.g. products with ≤1.5 grams of SFA per 100 grams of the product) between baseline and 3 months.  2. Changes in mean LDL-cholesterol, HDL-cholesterol, total cholesterol, non-HDL cholesterol, total cholesterol/HDL ratio and triglycerides between baseline and 3 months.  3. Changes in the intake of high SFA food groups between baseline and 3 months. |
| **Non-efficacy outcomes** | 1. Change in other components of diet  2. Other nutrient content of food purchases  3. Blood pressure  4. Body weight  5. Feasibility of the intervention and process measures  6. Qualitative sub-study | 1. 3-month changes in mean total energy intake, total fat, total sugars, fibre and salt between baseline and 3 months.  2. 3-month changes in mean energy density, total fat, sugar, fibre and salt from total purchases; mean total cost of the shopping basket (£) between baseline and 3 months.  3. Changes in mean systolic and diastolic blood pressure between baseline and 3 months.  4. Changes (absolute (kg) and relative (%)) in body weight between baseline and 3 months.  5. Feasibility of the intervention and process measures:  - Willingness of participants to take part in the study and be randomised to measure recruitment rates.  - Follow-up rates to measure programme attendance and retention.  - Acceptability of the intervention and other process measures.  6. Themes and sub-themes pertaining to the participants’ (i) knowledge, (ii) perceived barriers, facilitators and actions, (iii) contextual influences, and (iii) value of the healthcare advice provided. |

# ABBREVIATIONS

| AE | Adverse Event |
| --- | --- |
| BHF | British Heart Foundation |
| CI | Chief Investigator |
| CRF | Case Report Form |
| CTRG | Clinical Trials & Research Governance, University of Oxford |
| CVD | Cardiovascular Disease |
| GCP | Good Clinical Practice |
| GP | General Practitioner |
| HDL | High density lipoprotein cholesterol |
| HP | Health Professional |
| ICF | Informed Consent Form |
| LDL | Low density lipoprotein cholesterol |
| NHS | National Health Service |
| NICE | National Institute for Health and Care Excellence |
| NRES | National Research Ethics Service |
| PIL | Participant/ Patient Information Leaflet |
| R&D | NHS Trust R&D Department |
| REC | Research Ethics Committee |
| SFA | Saturated Fat |
| SOP | Standard Operating Procedure |

# BACKGROUND AND RATIONALE

Poor diet is a major contributor to cardiovascular disease (CVD). Saturated fat (SFA) increases the production of low-density lipoprotein cholesterol (LDL-C) and decreases its clearance from the body through suppression of the LDL receptor activity^1^. Meta-analyses of RCTs in which SFA has been replaced by unsaturated fats have estimated reductions of 10 to 17% in CVD events^2 3^, with particular improvements in vascular function produced by increases in monounsaturated fat^4^. However, progress in reducing SFA through public education programmes is slow, and SFA intake in the UK (13.5% energy) remains more than a third higher than the recommended value of <10% energy.

To identify effective interventions for LDL reduction through dietary improvements, we searched the Cochrane Database of Systematic Reviews, MEDLINE and the ISRCTN registry for ongoing studies and systematic reviews. The most recent systematic review and meta-analysis was published by the Cochrane Collaboration group in June 2015 and found that interventions to reduce SFA intake compared to usual diets resulted in a significant reduction in LDL cholesterol of 0.19 mmol/L [-0.33,-0.05] and 17% reduction in CVD risk^3^. The authors concluded that lifestyle advice should be given to those at risk of CVD and to lower risk population groups to achieve a meaningful reduction in saturated fat intake, which is consistent with the recommendations from NICE for CVD prevention^5^. However, while the evidence is clear about what should be done, it is far less clear that we have practical means of achieving these dietary changes in routine clinical practice.

Previous research has established that it is possible to achieve lower SFA intake through providing appropriate food stuffs in place of higher SFA products, in combination with intensive and tailored counselling^6 7^. However, the success of dietary advice alone to reduce SFA has been limited and has only been achieved with specialist staff and high intensity behavioural support^8 9^. It is increasingly recognised that the health system needs a mechanism to support a large number of people classified as at increased CVD risk. A recent small trial of dietary counselling in a primary care setting has shown a short-term beneficial effect on blood cholesterol^10^ but as yet there is no effective intervention that is sufficiently scalable and practical for routine delivery in primary care settings, or at population level.

Food purchasing is a key antecedent of food consumption and improving the nutritional quality of food purchases presents a clear opportunity to intervene. Supermarkets account for 71% of the weekly expenditure on food and drinks, including a large proportion of foods that are major sources of SFA in the diet such as meat, dairy, ready meals, cakes and biscuits^11^. Individual-level interventions targeting the nutritional quality of the grocery shopping could improve diet quality, especially among those motivated to change. However, previous evidence suggests that information provision alone might not be sufficient for sustained dietary change, and other behavioural strategies may be needed^12^.

Systematic reviews have identified effective intervention components for individual dietary change, including providing tailored dietary advice, information, self-monitoring and personalised feedback^13 14^. In-store education provision at the point of purchase has been shown to improve the nutritional quality of food purchases, and increase fruit and vegetable purchases^15^, and a previous intervention using an online supermarket showed a 10% reduction in SFA from food purchases by recommending lower SFA options at the point of purchase^16^. New technological advances within the food industry allowing the tracking of food purchases, including their nutritional content, offer the potential to deliver personalised nutrition interventions to reduce SFA intake. Here we plan to develop a programme to provide regular personalised feedback on the saturated fat content of food purchases, together with suggested alternatives with less saturated fat. To do so, we will establish a partnership with Tesco, the largest UK supermarket. Using a well-established storecard system, the store can track food purchases which can be linked to a nutritional database to calculate the nutritional content of the basket and, with further development, identify substitutes containing less saturated fat. The study will be run by the University of Oxford.  With participant consent, Tesco stores will provide data to the study team in order to enable the study to run. Tesco will not receive any additional information about participants as a result of the study.

Building on what is known but bearing in mind scalability, we propose to develop and test a novel intervention to decrease SFA intake. In one group, a brief advice session will provide motivational force using a qualified health professional to explain the importance of SFA intake and the impact this could make to a patient’s cholesterol. But recognising that health professionals may lack sufficient time and training to go beyond general messages about how to reduce SFA, we propose another intervention to provide personalised feedback and self-monitoring of dietary habits, which has further potential to achieve and sustain behaviour change^14^. Therefore, a second group will receive a brief advice session in combination with a personalised report on purchasing behaviours (provided monthly over the 3 months of follow up), to enhance the participants capability to make dietary changes when shopping for food. By drawing on the power of data already collected by supermarkets we are able to automate the process which would otherwise require considerable time from dietitians to deliver. These two groups will be compared to a control group which will just receive their blood test results without any further advice.

We will enrol people with raised LDL cholesterol who are willing to try to change their diet, perhaps to avoid the need for long-term medication. At this stage we will examine the effect of the intervention on saturated fat intake as the primary outcome and, if promising, we will plan for a definitive trial to test the effectiveness of the intervention to reduce LDL cholesterol. This proposed intervention is low cost and could reach large numbers, meaning that it could have a significant population impact and be very cost-effective, even if the effect size is smaller than more intensive interventions. If successful, similar interventions could be offered by supermarkets to all their customers to bring health benefits to the whole population and encouraging supermarkets to play a more proactive role in shaping healthier choices for their customers.

**Aims**

This project aims to develop and test a behavioural intervention to promote reductions in saturated fat (SFA) intake among patients in primary care with raised LDL-cholesterol who are willing to change their diet.

The main hypothesis is that compared to usual care, an intervention involving health professional advice alone and/or in combination with personalised feedback and monitoring on the SFA content of food purchases, will help people reduce dietary SFA intake which, in the longer term, would be expected to lead to reductions in LDL cholesterol.

Specific aims of this research include:

1. To develop an intervention comprising a brief advice session from a qualified health professional to promote healthier eating and reduce saturated fat;
2. In collaboration with a supermarket, to develop a system to provide personalised feedback on the nutritional content of the shopping basket, advice on alternative food options with lower SFA content, and tracking of the SFA content of food purchases over time;
3. To test the effectiveness of brief advice alone and the combined intervention in decreasing SFA intake among patients in primary care with raised LDL cholesterol.

# OBJECTIVES AND OUTCOME MEASURES

**Objectives**

i. Primary objective:

To develop and test the effectiveness of a behavioural intervention to reduce saturated fat (SFA) intake through brief advice from a qualified health professional alone or in combination with feedback on food purchasing behaviours.

ii. Secondary objectives:

To test the effects of the intervention on:

1. Other measures of dietary intake, including changes in mean total energy intake, total fat, total sugars, fibre and salt; and intake of high SFA food groups between baseline and 3 months.
2. Measures of food purchases including changes in mean %SFA from total purchases; energy density, total fat, sugar, fibre and salt from purchases; proportion of food items with low SFA (e.g. products with ≤1.5 grams of SFA per 100 grams of the product); mean total cost of the shopping basket (£) between baseline and 3 months.
3. Biochemical markers of CVD risk, including changes in mean LDL-cholesterol, HDL-cholesterol, total cholesterol and triglycerides; systolic and diastolic blood pressure between baseline and 3 months.
4. Body weight, including changes (absolute (kg) and relative (%)) in body weight between baseline and 3 months.
5. Feasibility of the intervention, including willingness of participants to take part in the study and be randomised to measure recruitment rates; follow-up rates to measure programme attendance and retention; and process measures such as the acceptability of the intervention for participants and health professionals and the uptake of the intervention.
6. Qualitative sub-study outcomes: Themes and sub-themes pertaining to the participants’ (i) knowledge, (ii) perceived barriers, facilitators and actions, (iii) contextual influences, and (iii) value of the healthcare advice provided.

**Outcome measures**

i. Primary outcome: change in mean SFA intake (% from total energy intake) between baseline and 3 months, measured using 2 x 24h dietary recalls twice (once at baseline and once at 3 months) using a validated web based questionnaire. We will measure intake at an individual level. We selected SFA intake as the primary outcome because this is the main target in most dietary interventions that aim to promote a healthier diet to prevent cardiovascular disease. Evidence from recent randomised trials in populations with high CVD risk showed absolute changes in %SFA intakes of around 4-5% when participants were provided with the appropriate food swaps^6 7^.

ii. Secondary and non-efficacy outcomes:

We will also examine changes in food purchasing patterns, including changes in mean %SFA from total purchases; energy density, total fat, sugar, fibre and salt from purchases; proportion of food items with low SFA (e.g. products with ≤1.5 grams of SFA per 100 grams of the product); mean total cost of the shopping basket (£) between baseline and 3 months. This is objective information collected through the storecard data, but available only at a household level. However, measures of the nutrients from the storecard data are expected to be highly correlated with nutrient intakes as captured by the dietary recalls but not perfectly if some participants buy food for themselves and different food for the rest of their household (i.e. only one in the household could be on a diet).

We will explore changes in other dietary intake measures using the same dietary instruments as for the main outcome, including changes in mean total energy intake, total fat, total sugar, fibre and salt, as well as changes in the intake of high SFA food groups between baseline and 3 months. Since the intervention will propose specific healthier swaps to decrease total SFA intake, changes in some of these other nutrients and food groups can also be expected, particularly to energy intake and total fat, or there may be compensatory increases in other nutrients such as sugar.

Changes in the biochemical markers of CVD risk will be also examined, including LDL-cholesterol, HDL-cholesterol, total cholesterol, non-HDL cholesterol, total cholesterol/HDL ratio, triglycerides, systolic and diastolic blood pressure between baseline and 3 months. We selected these biomarkers as they are the prime targets for CVD risk reduction and have improved in previous interventions that provided appropriate food swaps to reduce SFA intake^6 7^. A recent systematic review and meta-analysis found that interventions to reduce SFA intake compared to usual diets resulted in a significant reduction in LDL cholesterol of 0.19 mmol/L [-0.33,-0.05] and 17% reduction in CVD risk^3^.

Changes in body weight (absolute (kg) and relative (%)) will be examined between baseline and 3 months. Although the intervention will only suggest food swaps with lower SFA content, many of these foods are high in energy density and swapping to lower fat intake generally reduces energy density and total energy intake. Hence it is expected that participants in the active intervention arms will decrease their total energy intake and small changes in body weight could be observed as has happened in other behavioural interventions targeting the nutritional quality of the diet in patients with high CVD risk^7^.

We will also assess key elements of feasibility and process measures. These include willingness of participants to take part in the study and be randomised, which will help determine recruitment rates; programme attendance and retention, which will help determine follow-up rates. Finally, we will measure the acceptability of the intervention for participants and health professionals, and process measures will be collected using questionnaires before, and after the intervention period. These include questions to understand if the given information (provided by the BHF leaflet and the purchasing reports) was helpful and clear for the participants and improved their knowledge and motivation to change; if the purchasing report was helpful to them in subsequent shopping episodes and to track their progress; if the report was easy to understand and provided acceptable alternatives with lower SFA; if the frequency of the purchasing reports was adequate; if the purchasing report enhanced their motivation to select items with less SFA, and if the perceived cost of their shopping is unchanged after the intervention. The questionnaire will also assess attitudes towards diagnosis and treatment of dyslipidaemia and cardiovascular disease, and the value of their health care provider offering support to change their diet. Furthermore, uptake of the intervention will be objectively measured by using information on participants’ purchases before and after the intervention to understand if the suggested swaps by the BHF leaflet and/or the purchasing report were accepted and people truly changed their shopping behaviours. Finally, we will also record health professionals’ views and feelings about delivering the brief advice session to improve diet and decrease SFA intake for the participants in the study.

Qualitative study: *A priori* hypotheses and/or outcome measures are not specified. Rather, a deductive thematic analysis approach will draw out themes and sub-themes pertaining to the participants’ (i) knowledge and health values about SFA (ii) perceived barriers and facilitators and actions, (iii) contextual influences that may have affected the overall result, and (iii) value of the healthcare advice provided to motivate and support individuals to change their food purchasing behaviour.

| **Objectives** | **Outcome Measures** | **Timepoints of evaluation of this outcome measure** |
| --- | --- | --- |
| **Primary Objective**  To test a behavioural intervention to promote reductions in saturated fat (SFA) intake through brief advice from a qualified health professional alone or in combination with feedback on food purchasing behaviours | Change in SFA intake, measured using 2 x 24h dietary recalls using the Web-Q instrument. | Mean % SFA intake at baseline and 3 months. |
| **Secondary Outcomes**  1. Purchasing patterns  2. Biochemical markers of CVD risk  3. Food group patterns | 1. Changes in SFA from total purchases; proportion of food items with low SFA (e.g. products with ≤1.5 grams of SFA per 100 grams of the product);  2. Changes in LDL-cholesterol, HDL-cholesterol, total cholesterol and triglycerides;  3. Changes in the intake of high SFA food groups using 2 x 24h dietary recalls. | 1. Mean %SFA from total purchases; proportion of food items with low SFA at baseline and 3 months.  2. Mean LDL-cholesterol, HDL-cholesterol, total cholesterol, non-HDL cholesterol, total cholesterol/HDL ratio and triglycerides at baseline and 3 months.  3. Mean intake (g, kcal) of high SFA foods at baseline and 3 months |
| **Non-efficacy Outcomes**  1. Intake of other nutrients  2. Other purchasing patterns  3. Blood pressure  4. Body weight | 1. Changes in energy intake, total fat, total sugars, fibre and salt using 2 x 24h dietary recalls.  2. Changes in energy density, total fat, sugar, fibre and salt from purchases; mean total cost of the shopping basket (£).  3. Changes in systolic and diastolic blood pressure.  4. Changes (absolute (kg) and relative (%)) in body weight. | 1. Mean total energy intake, total fat, total sugars, fibre and salt at baseline and 3 months.  2. Mean energy density, total fat, sugar, fibre and salt from purchases; and mean total cost of the shopping basket (£) at baseline and 3 months.  3. Mean systolic and diastolic blood pressure at baseline and 3 months.  4. Mean body weight at baseline and 3 months. |
| **5. Feasibility and Process Measures**   - Recruitment rates - Follow-up rates - Acceptability and views of the intervention; and other process measures | - Willingness of participants to take part in the study and be randomised. - Programme attendance and retention. - Acceptability of the intervention for participants and health professionals; uptake of knowledge, motivation and swaps accepted. | - Number of participants who accept the invitation, consent to take part in the study and are randomised at baseline - Number of participants who return for the follow up visit at 3 months - Questionnaires at baseline, 3 months and immediately after the intervention is delivered. Number and types of swaps purchased during the intervention. |
| **Qualitative Sub-study** | To conduct a semi-structured, one-to-one, telephone interview with a sub-sample of the PC-SHOP study participants to explore issues related to food purchasing behaviours, including knowledge, perceived barriers, facilitators and actions, contextual influences, and value of the healthcare advice provided. | Themes and sub-themes pertaining to the participants’ (i) knowledge, (ii) perceived barriers, facilitators and actions, (iii) contextual influences, and (iii) value of the healthcare advice provided. |

# STUDY DESIGN

For this study, an individually randomised, 3 arm, parallel group design will be employed.

Due to the nature of the intervention, it will not be possible to blind participants, clinicians or some of the study team to the treatment allocation once the intervention commences, but they will be blind at the point of randomisation.

Patients’ participation in the study will last 3 months from randomisation to final follow-up. Details of the sequence and duration of all study periods are included in the appendix together with the flowchart of the project.

Briefly, participating practices will search their records for eligible participants who meet the essential inclusion criteria: a) adults and b) with a record of high LDL cholesterol over the last 24 months. Invitation letters will be sent with the team contact information and study details, including other inclusion criteria a) participant is primarily responsible for the household grocery shopping and b) participant shops at Tesco at least once a week using a Tesco storecard.

Interested participants who believe they meet the criteria will be invited to contact the central research team via email who will assess them further over the phone to check eligibility. Those who meet all the eligibility criteria will be booked for the first baseline visit. Previous to the first baseline visit, participants will be asked to provide the first 24h dietary recall (using a validated online questionnaire performed on a computer). This data will be stored independently of the study team before full consent is obtained.

At the baseline visit participants will provide full informed consent and a blood sample by fingerprick will be taken by the central research team. Additionally, the central research team will collect other baseline measures, including a second 24h dietary recall, weight and height, blood pressure and their Tesco’s clubcard number. Once the blood tests confirm that each participant has raised LDL cholesterol and that they have completed two dietary recalls, the central research team will randomise them to one of the three interventions. Those allocated to brief advice session or brief advice session with purchasing report will be booked for an appointment with the health professional at their practice within a week after the baseline visit preferably. Those allocated to the control arm will receive usual care, which consists of providing their blood results.

All participants will come back after 3 months for a third visit (follow up) in which some outcome measures collected at baseline will be repeated, including the blood sample, the 24h dietary recalls, weight, blood pressure, and a questionnaire to assess acceptability of the intervention. Additionally, at the end of the study period, all participants will receive a purchasing report regardless of their treatment group allocation as well as the results of their final blood test.

# PARTICIPANT IDENTIFICATION

## Study Participants

The study population will include 112 adults ≥18 years of age with confirmed high LDL cholesterol (>3 mmol/L) at baseline, who will be recruited and randomised to intervention or comparator groups.

Screening and recruitment will be performed locally through primary care practices to identify eligible individuals. Screening criteria will be: age ≥18 years with a blood test in the GP records which indicate LDL-cholesterol >3.5 mmol/L (or total cholesterol >5.5 mmol/L if an LDL measure is not available) in the past 24 months, who will likely benefit from a lower SFA diet with no pre-existing conditions that warrant exclusion.

Participating practices will search their records to find people who meet the screening criteria and will send invitation letters explaining the benefits of joining the study and the contact information to take part in the study. Patients whose GP judges not able to meet the demands of the study or unlikely to adhere to study procedures as stated in the protocol will not be invited to take part in the study. All eligible participants will have to have a blood test to confirm that they have high LDL at the moment of recruitment to be eligible for the study.

## Inclusion Criteria

- Male or Female, aged 18 years or above.
- Express a desire for support to improve the nutritional quality of their diet to reduce their CVD risk.
- Primarily responsible for household shopping (e.g. complete at least half of their household shopping).
- Shops mainly at Tesco (e.g. at least once/week instore and/or online) using a Tesco’s storecard.
- Have had a Tesco’s storecard registered exclusively under their name for at least 3 months before recruitment.
- Computer literate (e.g. use email regularly and is able to perform dietary questionnaires online).
- Participant is willing and able to give informed consent for participation in the study.
- With confirmed LDL cholesterol above 3 mmol/L at recruitment.

## Exclusion Criteria

- Unable to read and understand the instructions provided in English.
- Pregnant, or planning to become pregnant during the course of the study.
- Started cholesterol-lowering medication in the last 3 months.
- Planned changes to cholesterol-lowering medication in the next 3 months.
- Existing cardiovascular conditions: heart attack or stroke or new diagnosis of atrial fibrillation within the last 3 months; heart failure of grade II New York Heart Association and more severe, or prolonged QT syndrome, angina, Arrhythmia, or familial hyperlipidaemia.
- Currently or recently (within the last 3 months) participating in another intervention study which likely affects the outcomes measured in this study.
- Patients that the GP judges not able to meet the demands of the study or unlikely to adhere to study procedures as stated in the protocol.

# STUDY PROCEDURES

An overview of the study procedures is shown in the study flowchart (Appendix A).

## Screening and Recruitment

Participating practices will be asked to search their electronic health records to screen suitable participants for the study. We will invite practices to participate who have access to a dietary counselling service by a nurse or qualified health professional which can assist with the intervention. As a result of the search, eligible patients will be sent an invitation letter from their GP as part of a staggered mail out. Participants will be provided with contact information and will be encouraged to contact the research team via email if they are interested in taking part and meet all the inclusion criteria.

Prior to the invitation letters being sent, the GP will screen the search list to ensure that all those identified are medically appropriate to participate in the trial. The GP will exclude those that it would be inappropriate to include according to the specified exclusion criteria and those not able to meet the demands of the study or unlikely to adhere to study procedures as stated in the protocol.

## Eligibility Assessment

Participants who are interested in taking part will contact the central research team on the email provided to them in their letter from the GP. The central research team will discuss study participation with potential participants (by telephone) and undertake eligibility assessment. If the potential participant appears eligible and would like to attend a baseline assessment to confirm eligibility, they will be offered an appointment at their GP practice.

The central research team will post a Participant Information Sheet (PIS) and confirmation letter. Participants will be asked to complete the first 24h dietary recall before the first baseline visit. This data will be stored independently of the study team before full consent is obtained. Participants will be also informed that any data provided before the baseline visit will be destroyed if they decide not to continue taking part in the study, are found to be ineligible or they don’t attend the baseline visit.

When the participant attends the practice, the central research team will provide information as described in the PIS to seek informed consent and will check eligibility for inclusion in the study by assessing the inclusion and exclusion criteria as described above; and confirming elevated LDL cholesterol with the blood sample taken at this baseline visit.

## Informed Consent

The central research team will explain the demands of the trial and answer potential participants’ questions. It will be clearly stated that the participant is free to withdraw from the study at any time for any reason without prejudice to future care, without affecting their legal rights, and with no obligation to give the reason for withdrawal.

The participant will be allowed as much time as wished to consider the information, and the opportunity to question the Investigator, their GP or other independent parties to decide whether they will participate in the study. Evidence of consent will be obtained in writing by means of participant dated signature and dated signature of the person who presented and obtained the consent. The person who obtained the consent will be suitably qualified and trained in Good Clinical Practice procedures, and have been authorised to do so by the Chief Investigator. A copy of the signed consent form will be given to the participant and a copy will be given to the participant’s GP. A scanned copy will be sent to Tesco. The original signed form will be retained by the central research team.

Since the intervention involves providing participants with a personalised report on their previous shopping at Tesco supermarkets, consent to access the storecard data will be obtained by the central research team. Tesco's team will be given the name and storecard number of the participants in the study in order to arrange data transference between Tesco and the central research team. Consent will be sought from participants to allow this and a copy of the consent form will be given to Tesco. Tesco will not receive any additional information about participants as a result of the study.

## Randomisation, blinding and code-breaking

Once the blood test taken during the first baseline visit confirms that the participant has raised LDL cholesterol (>3 mmol/L) and after confirming that they have completed a second 24h dietary recall (details in the next section), and provided their Tesco’s storecard details, the central research team will randomise all eligible participants (until we reach our target sample size) with an allocation ratio of 1:3:3 to control or one of the two active interventions using the online RedCap program which ensures full allocation concealment as information on future allocations are not accessible to the person randomising. Allocation will use block randomisation with randomly varying block sizes of size 7. Once randomised, participants will be booked for a second baseline visit in which they will receive their intervention depending on the group they have been allocated to.

Due to the nature of this trial, it will not be possible to blind participants, clinicians or some of the central research team to the treatment allocation. Nevertheless, the primary outcome will be a measure of saturated fat intake collected through a web-based questionnaire which individuals will fill in individually without any involvement from the study team. The brief advice intervention will be delivered by the practice nurse, who won’t be aware of the treatment allocation within the active intervention arms.

## Baseline Assessments

The baseline assessment will take place at the first appointment in which the central research team collects informed consent and performs eligibility assessments, unless participants request additional time to consider their participation in the study.

The central research team will collect the following measurements at the practice:

- Fasting fingerprick blood sample, to be analysed for blood lipids (LDL, HDL, total cholesterol, non-HDL cholesterol, total cholesterol/HDL ratio and triglycerides) using a portable lipid testing system, will be taken by the central research team. This blood sample is needed to confirm elevated LDL cholesterol >3 mmol/L,
- Demographic (age, gender, ethnicity and education, number of people in the household), health behaviours (alcohol and smoking), shopping habits and prior knowledge in regards to diet and SFA collected by questionnaire,
- Relevant medical history and all concomitant medication,
- Dietary intake will be collected using a validated web-based 24-h recall dietary questionnaire, which will be completed twice at baseline, at the in-person visit and before the visit at the participant’s home using their own computer. Participants will be given the opportunity to come to the practice to complete their dietary recall if they are unable to access a computer and internet at home,
- Seated systolic and diastolic blood pressure,
- Weight and height.

Participants will also provide their storecard number, which will be used by Tesco to provide their shopping information during the study period.

Each of these measurements and procedures will be detailed in the study manual held at each site.

## Subsequent Visits

The aim of subsequent visits is to assess the efficacy and non-efficacy outcomes of the trial.

Scheduling of follow-up visits is the responsibility of the central research team, who will contact participants to schedule the follow-up assessments. We will make at least three but no more than five attempts, to contact participants by telephone. We will then use other means (letter; email). Additionally, the central research team will ask the participant to complete one 24h dietary recall prior to the follow up appointment.

## Three-month follow up assessment

A follow up and final visit will be completed at least 3 months and no more than 4 months after randomisation to assess the main study outcomes. Therefore, all the measures collected as part of the baseline visit will be repeated following the same procedures and conditions as described above.

The central research team will collect the following measurements at the practice:

- Fasting fingerprick blood sample, to be analysed for blood lipids (LDL, HDL, total cholesterol, non-HDL cholesterol, total cholesterol/HDL ratio and triglycerides) using a portable lipid testing system, will be taken by the central research team,
- Any changes in knowledge with regards to diet and SFA as well as intervention acceptability collected by questionnaire,
- Changes to relevant medical history and all concomitant medication,
- Dietary intake will be collected using a validated web-based 24-h recall dietary questionnaire, which will be completed twice at follow up, once prior to the follow up visit, and a second one at the in-person visit. Participants will be given the opportunity to come to the practice to complete their dietary recall if they are unable to access a computer and internet at home,
- Seated systolic and diastolic blood pressure,
- Weight.

- Changes to drug treatments during the course of the study: the clinical team in the practice will decide which, if any medications need to be adjusted as a result of changes in health status during the course of the study. However, we will ask participants and clinicians to refrain from altering any lipid-lowering medication during the 3-months of follow-up unless strictly necessary.

## Food shopping data

The proposed intervention involves providing participants with a personalised report on their previous shopping at Tesco supermarkets, therefore a partnership has been established with Tesco to obtain participants’ consent to access the loyalty card data. However, if Tesco is unable to fully meet the expectations of this partnership and cannot provide information in “real time” (e.g. before randomization), a contingency plan has been developed to fill this gap. We will ask participants to collect and store their till receipts from Tesco during a baseline period of 4 weeks before randomization and over the intervention period. We will ask participants to provide us with copies of their receipts at the baseline visit and post them to the study team during the intervention period if access to the loyalty card data is not possible. This will allow us to manually code their shopping and generate their feedback report to show “proof of concept” that generating feedback based on previous purchases would be helpful to improve dietary choices.

## Sample Handling

Fingerprick blood samples will be handled, analysed and then disposed as per protocol using a portable lipid testing system. Blood will be taken at baseline and 3 months to estimate changes in biomarkers of cardiovascular disease risk (total, LDL and HDL cholesterol, non-HDL cholesterol, total cholesterol/HDL ratio, triglycerides).

## Alternative scheduling for follow-ups

The central research team will conduct follow up visits at the participant’s home if they agree and the participant is unwilling to attend the practice. If it’s not possible to do in-person visits, we will record self-reported measures of primary and secondary outcomes by telephone, text or email, if possible.

## Participant reimbursement

As a result of participation in the PC-SHOP trial participants will be asked to attend 2 (control) or 3 (intervention groups) study visits at their local GP practice. In order to minimise the burden on participants of claiming travel expenses (providing receipts, recording mileage, completing forms) the research team will offer each participant a £10 gift card at the 3-month study visit.

## Discontinuation/Withdrawal of Participants from Study

Each participant and their GP have the right and can ask to withdraw from the study at any time. In addition, the Chief Investigator may discontinue a participant from the study at any time if they consider it necessary for any reason, including for example, ineligibility (either arising during the study or retrospectively having been overlooked at screening).

If a participant (or their GP) requests to withdraw from the study, we will explain that we would like to use their data up to the point that they have withdrawn, unless they request that we do not do so. The reason for withdrawal will be recorded in the CRF.

Withdrawn participants will not be replaced.

## Definition of End of Study

The end of study is the date the last participant has their last data capture for the 3 month follow-up visit.

## Qualitative sub-study

This qualitative study aims to conduct a qualitative process evaluation to examine the perceived value of the behavioural change techniques used in the intervention and the perceived impact on food purchasing behaviours through semi-structured, one-to-one, telephone interviews with approximately 28 participants in intervention 2 group.

This qualitative study will play a valuable role as part of the process evaluation of the trial and will help in the interpretation of the findings of the trial. If the trial outcome is promising it may point to areas that could be enhanced ahead of any future definitive trial to test the effectiveness of the intervention to reduce LDL cholesterol.

**Recruitment**

Once the main study has finished follow up, we will start recruitring participants from the intervention 2 arm of the PC-SHOP study and who are willing to be interviewed. Forty-five participants are potentially eligible and we will continue to recruit until sufficient numbers and data saturation are achieved, estimated based on experience to be approximately 28 people.

First, participant details (including their postal and email address, and intervention arm they were assigned to) will be accessed via the PC-SHOP intervention study electronic, password encrypted database. This database holds all identifiable data and is accessible by the research team only.

A member of the research team will mail all participant’s (i.e. those in intervention 1, intervention 2 and controls) their blood results, which were obtained as part of their final PC-SHOP study visit. This mailing will also include an invitation letter to be telephone interviewed by a member of the research team. Invitations letters will only be sent to participants who participated in intervention 2.

Interested participants will be encouraged to call or email the PC-SHOP study team to ask further questions and either accept or decline the invitation. If a participant accepts the invitation, a convenient date and time for the telephone interview will be arranged and the participant information sheet (PIS) will be sent. Participants will be free to withdraw at any time prior to and during the interview. Non-respondents will be emailed up to two times via the PC-SHOP shared mailbox [pcshop@phc.ox.ac.uk](mailto:pcshop@phc.ox.ac.uk) after their initial postal invitation to remind them before ceasing to try to make contact. We will continue to recruit until data saturation has been reached or when all participants who are willing have been interviewed.

Data saturation is the point when no new themes emerge during analysis. In line with qualitative methods for data collection, we use a zig-zag approach involving the simultaneous process of data gathering and coding meaning the planned sample size (*N*~28) may either increase or decrease depending on when data saturation is reached. Terminating participant recruitment at the point of data saturation will prevent data over collection.

**Consent**

A member of the research team (either research assistant [RA] or chief investigator [CI]) will facilitate the telephone interview (hereafter referred to as researcher). They will have received training in good research governance and Good Clinical Practice (GCP) no more than two years prior to the interview.

On the day of the interview, but prior to commencement, the researcher facilitating the interview will explain the aim and demands of the telephone call and answer the participant’s questions. The researcher will then explain that the telephone call will be recorded, and invite the participant to agree to the audio recording. If the participant agrees, the researcher will press record. If the participant declines, the researcher will end the interview and provide a full explanation as to why.

Next, the researcher will invite the participant to give verbal informed consent. Each consent statement of the consent form will be read verbatim by the researcher, to which the participant will be invited to verbally agree by replying ‘*yes*’. These consent statements will include an explanation that their data will be held confidentially under the Data Protection Act 2018 and that they may withdraw from the interview at any time without implications for their standard health care, without affecting their legal rights, and with no obligation to answer further questions or state the reason for withdrawal. The letter invitation and PIS will also explain the right to withdraw. Each statement numbered 1 to 7 are required statements (i.e. non-optional) and failure to provide informed consent on one or more will terminate the participant’s involvement in the study.

The researcher will initial each point to indicate verbal consent was provided. Since the verbal consent will be audio-recorded it will also be transcribed verbatim. The research team at the sponsor site will securely retain both original copies of the researcher-initialled and verbally transcribed consent transcript. The RA will post copies of the researcher-initialled consent form to the participant.

The PIS contains the Oxford University Hospitals (OUH) Patient Advice and Liaison Service (PALS) contact details should the participant wish to discuss the interview with an independent party outside of the central research team.

**Interview**

**Telephone Call (30 minutes)**

The researcher will telephone call participants on the arranged date and time. Telephone calls will be made in a pre-booked, quiet room at the University of Oxford. They will be audio-recorded and last approximately 30 minutes.

First, participants will be required to confirm their name. Next, the researcher will remind participants of the PC-SHOP intervention study. The researcher will describe the overall structure of the telephone call and provide an opportunity for the participant to ask questions.

The researcher will then explain that the telephone call will need to be recorded, invite the participant to provide consent for audio recording and then press record accordingly. The participant will be invited to provide verbal informed consent. The researcher will read a series of seven required statements pertaining to voluntary participation, data collection and management, and anonymisation and confidentiality. The participant will be required to reply ‘*yes*’ if they consent. If the participant declines consent to one or more statements, they will be thanked for their time and receive and the telephone call will end. The researcher will explain why. These participants will still receive the store card reimbursement.

Consented participants will be asked around 10 questions pertaining to the following themes: (1) understanding of saturated fat and the effects on health (2) the intervention components and impact on behaviour, and (3) support from healthcare professionals to improve health. For example, “*what did you think about healthier swaps suggested?*” (Question 7). All members of the PC-SHOP research team have developed and piloted the topic guide. In line with qualitative research methods, minor edits to the topic guide may be made during the data analysis if unexpected themes begin to emerge.

At the end of the interview, the researcher will provide participants with the opportunity to add any other thoughts. They will remind participants to expect a letter in the post with the researcher-initialised consent form and a £20 store card as reimbursement for their time. The researcher will then stop the audio recording.

**Audio Transfer to Secure Storage**

The Information Asset Registrar form defines this study’s audio recordings as an asset. Hence, all audio recordings will be uploaded to the University of Oxford’s electronic secure storage facility accessible only by the research study team. These audio recordings will be transformed into an encrypted zip file using 7 Zip. The research team will then store recorders (as well as all study data documents, including the consent form) securely in a locked cabinet, in a locked room within a restricted access building with keypad access control system.

**Audio Transcription**

The encrypted zip files will be sent for transcription via OxFile to approved external transcribers, with confidentiality and conduct contracts in place with the University of Oxford. The process for transferring the zip files will be performed according to local Information Governance policy; in password-protected files, with the agreed password transferred via a separate form of contact i.e. different email address or telephone number. The external transcriber shall transfer transcriptions back to the researcher via OxFile. The original audio files will be deleted from the recorder once the transcripts have been checked for fidelity and any queries resolved. Once transcripts have been checked, the study team will de-identify them (e.g. deleting any references to identifiable information such as names) before they are stored. Transcriptions will be stored anomymously in the electronic secure storage facility, backed up, and archived using the University of Oxford Hierarchical File Server (HFS) Backup service.

**Analysis of the qualitative study**

Verbatim transcriptions will be analysed using the NVivo 11 software programme^4^. A thematic analysis approach will draw out themes, categories and nodes from the data pertaining to the (i) knowledge, (ii) perceived barriers and facilitators, (iii) contextual influences, and (iii) value of the healthcare advice provided. One researcher will deductively code the transcript data against an initial thematic framework using the Framework Method^5^, which comprises five steps: (i) familiarisation, (ii) developing a thematic framework, indexing, charting, mapping and interpretation.

A second researcher will check a sub-sample of 10 percent for validity and inter-rater reliability. In instances where it is unclear how to code themes against the initial framework, discussion with a third reviewer will reach consensus on whether to form a new theme or whether to expand an existing theme.

Thematic coding will begin once data collection starts in a process of iterative data gathering and analysis i.e. a zigzag approach. Early analysis will inform further data gathering. For this reason, the topic guide will be subject to ongoing review and edits if unexpected themes begin to emerge. Findings will be synthesised narratively.

# INTERVENTIONS

The two active interventions will incorporate strategies which use the Behaviour Change Wheel COM-B model as a theoretical framework to guide the choice of behavioural strategies^13 17^. Briefly, the proposed interventions aim to allow participants to develop and sustain the capability, opportunity and motivation to make changes to diet^13^. Psychological capability will be enhanced by educating participants on SFA and health risks as well as information on the most appropriate swaps to decrease their SFA intake. Opportunity will be supported by the shopping report which will highlight the top 3 individual sources of SFA and proposed swaps to those, therefore making healthier choices easier to spot by participants. Motivation will be promoted by the shopping report by allowing participants to track and monitor their progress over time, as well as by the health professional advice which will provide further motivation to attempt the change and facilitate understanding of the value of these changes on health.

Participants will be randomised to one of the three conditions explained below. Participants randomised to receive intervention 1 and 2 which involves a brief advice session, will be asked to consent to have the session audio-recorded to allow for fidelity testing. The purpose of fidelity testing is to determine:

- If the brief advice session in being delivered according to the intended protocol and behavioural theoretical models.

- If number frequency, length and content of the sessions are similar across all participants within each group.

- Compare and identify successful and unsuccessful aspects of the interventions.

## Intervention 1: Brief advice session

This intervention will be a single one-to-one appointment with the health professional (HP) in which they will receive advice on the importance of dietary change and motivation to reduce SFA based on the British Heart Foundation “*Cut the Saturated Fat*” chart^18 19^, which will be provided as printed educational material.

This session is aimed to inform participants about the benefits of saturated fat reduction and encourage them to attempt it. Dietary advice will be particularly focused on explaining the different sources of fat and the most appropriate changes proposed in the BHF guidance to decrease SFA, primarily by substitution with lower SFA options (e.g. change from regular beef to lean beef); or with mono- and polyunsaturated fat sources (e.g. change from butter to low-fat vegetable spread).

Health professionals will be trained to deliver this session face-to-face during approximately 10min. The structure of the session will be standardised so that it will follow 3 major discussion points. The HP will start by discussing cardiovascular risk with the participant and will provide the participant with the results of the blood tests. The HP will then highlight that diet is a very important modifiable risk factor which can potentially help them decrease this risk by lowering their blood lipids. The second point of discussion will focus on the major sources of SFA in different foods. The HP will carefully explain the educational materials with special emphasis on the major sources of SFA in the UK (dairy, meat and cakes/biscuits). The session will end with a few minutes for questions and final remarks in which the participant can ask any questions in regards to the information provided and the HP will finish the session by emphasising that the proposed changes can achieve very good results in a short time period.

## Intervention 2: Brief advice session and shopping report

In addition to the brief advice session detailed above, participants in this intervention arm will receive a personalised report with the nutritional composition of foods and beverages purchased from Tesco over the previous 3 months (baseline) and subsequent months (monthly over the 3 months of follow up). This report will include information on total saturated fat (g) and the major food contributors to SFA. Participants will receive monthly reports to allow them to chart their progress in reducing SFA in their grocery purchases. This information will be accompanied by a list with suggestions for one-for-one swaps for foods high in SFA for lower SFA foods to support dietary change.

## Control group

Participants in the control group (usual care) will be recruited into the study and will be informed of their blood tests by the study team. They will be seen again at 3 months for collecting outcome measures.

Upon completion of the study, all participants including those in the control group will receive a final report with the nutritional information of their purchases over the 3 months during which they were enrolled in the intervention. The control group will also be sent the BHF booklet.

# SAFETY REPORTING

This intervention will only provide standard dietary advice which is consistent with recommendations from scientific advisory committees in the UK and internationally and there is no known risk from eating food lower in SFA. Due to this, we will only report serious adverse events that the PI believes are related to the trial, which will be considered unexpected.

## Definition of Serious Adverse Events

A serious adverse event (SAE) is any untoward medical occurrence that:

- results in death
- is life-threatening
- requires inpatient hospitalisation or prolongation of existing hospitalisation
- results in persistent or significant disability/incapacity
- consists of a congenital anomaly or birth defect.

Other ‘important medical events’ may also be considered serious if they jeopardise the participant or require an intervention to prevent one of the above consequences.

NOTE: The term "life-threatening" in the definition of "serious" refers to an event in which the participant was at risk of death at the time of the event; it does not refer to an event which hypothetically might have caused death if it were more severe.

## Reporting Procedures for Serious Adverse Events

The duration of the SAE recording period lasts from enrolment on to the study to the end of the termination of their programme. All SAEs, deemed related by the PI, will be recorded at the time the central research team become aware of the incident. This may occur at study assessment visits, via notification from the clinical team or when the central research team contact participants by telephone or email.

The following information will be recorded for each SAE:

- Description,
- Date of onset and end date,
- Severity,
- Action taken,
- Follow-up information should be provided if deemed clinically necessary.

If there are any unexpected related SAEs, these will be reported to the Research Ethics Committee in accordance with HRA process.

# STATISTICS AND ANALYSIS

## Description of Statistical Methods

The primary statistical analysis of efficacy outcomes will be carried out on the basis of intention-to-treat (ITT). We will endeavour to obtain full follow-up data on every participant to allow full ITT analysis, but we will inevitably experience the problem of missing data due to withdrawal, loss to follow up, or non-response to questionnaire items.

We will analyse the primary and secondary outcomes with a linear regression model with adjustment for practice and baseline values. We will assess the sensitivity of the analysis to different assumptions about missing data using two imputation methods commonly used: completer only analysis and baseline observation carried forward.

The results from the trial will be prepared as comparative summary statistics (difference in means) with 95% confidence intervals. All the tests will be done at a 5% two-sided significance level. The study results will be reported in accordance with the CONSORT (Consolidated Standards of Reporting Trials) 2010 statements. A full detailed analysis plan (including plans for any interim analysis, subgroup analysis, and sensitivity analysis) will be prepared and finalised before any data analysis.

## The Number of Participants

The total number of participants we intend to recruit for this study is 112, with a sampling ratio of 1:3:3. Therefore, 16 participants will be allocated to control, and 48 participants will be allocated to each of the two active interventions.

Previous studies such as highly controlled interventions in populations with high CVD risk have achieved absolute changes in %SFA intakes of around 4-5%^6 7^, and a short-term change in LDL-cholesterol of 0.32 mmol/L by a low-intensity primary care-based intervention^10^. Attrition rates have been reported to be quite low in short term primary care-based trials^10 20^, so we could assume around 10% attrition rate in our study. In order to detect a reduction of 3% in %SFA intake (3% standard deviation) between each intervention and control with 90% power and two-sided α=0.05 using intention-to-treat analyses, we will need 16 participants in the control group and 48 in each intervention arm to account for 10% attrition and multiple comparisons. By recruiting 48 participants in each active intervention arms, we will be able to detect a further difference between these two arms of 2% (3% SD) with 90% power.

In addition, this sample size will allow to detect a change in LDL cholesterol of 0.3 mmol/L (SD 0.8) between each intervention and control with 60% power using a 2-sided α=0.05.

## Analysis of Outcome Measures

As described above, primary and secondary outcomes which are continuous variables will be analysed using linear regression models in an ITT analysis as the primary statistical analysis.

The primary analysis will test for:

- A difference in the change (from baseline to follow up) in % SFA intake between each intervention group compared to control;

- A difference in the change (from baseline to follow up) in % SFA intake between the two active intervention arms.

Using the appropriate interaction terms in the above mentioned models, we will perform subgroup analyses of the primary and secondary outcomes by sociodemographic characteristics (SES), as a previous study has shown that lower SES households purchase a higher proportion of energy from less healthy foods with smaller but important differences in the SFA content from purchased foods which may lead to differential effectiveness^21^. While the study is not powered to look for such effects here, this information will inform planning of subsequent research.

Acceptability of the intervention by the participants will be collected by questionnaire and will be summarised by presenting the frequencies of each response in bar charts.

# DATA MANAGEMENT

## Access to Data

Direct access will be granted to authorised representatives from the Sponsor and host institution for monitoring and/or audit of the study to ensure compliance with regulations.

## Data Recording and Record Keeping

We have discussed the data management and quality assurance of this study with a Senior Trials Manager from the UKCRC registered Primary Care Clinical Trials Unit (CTU). We agreed that they will provide general advice and support to ensure we follow CTU best practice but this study will not formally be adopted in the CTU portfolio. A study specific Data Management Plan (DMP) will be developed for the trial outlining in detail the procedures that will be put in place to ensure that high quality data are produced for statistical analysis.

A secure online database (password protected and kept on secure servers) will incorporate an electronic Case Report Form (CRF) onto which the central research team will enter data. If a paper CRF is needed, the original CRFs will be kept and returned by the central research team to the University of Oxford research base on a lockable briefcase and a copy will be held at the research site. All CRFs (electronic or paper) will be date stamped upon receipt. The central research team will store all paper CRFs and other study data documents, including consent forms, securely at the Primary Care department in a locked cabinet, in a locked room within a restricted access building with keypad access control system. A full pre-entry review and electronic data validation for all data entered into the clinical database will be provided by study specific programmed checks. Data from the dietary questionnaires will be collected and stored independently from the study team by the Cancer Research Unit servers (Nuffield Department of Population Health, University of Oxford) in anonymised form (using a unique study ID for each participant), and will be transferred to the study team only after full consent has been obtained using an encrypted and secure system. If a participant declines consent or is found to be ineligible, their questionnaire data will be deleted by those holding it independently.

On completion of the trial and data cleaning, the study documentation will be transferred to a secure, GCP compliant archiving facility, where they will be held for 5 years. Prior to database lock, a dataset review will be undertaken by the Chief Investigator and the trial statistician.

## Study Management

The Chief Investigator will be responsible for project coordination, and will be the lead trial manager and data analyst. She will be supported by Prof. Susan Jebb and Prof. Paul Aveyard who will provide expertise in development of complex behavioural interventions in primary care and trial design. A Senior Trial Manager from the UKCRC registered PC-CTU will provide further advice and support to the Chief Investigator throughout the study in relation to trial management and will oversee the operational aspects of the trial. A trial statistician will review and approve the statistical analysis plan and will provide support for the statistical analysis.

# QUALITY ASSURANCE PROCEDURES

## Quality Control and Quality Assurance Procedures

Regular monitoring will be performed. Data will be evaluated for compliance with the protocol and accuracy in relation to source documents where possible. Following the standard procedures specified in the protocol and working instructions, the study members in charge of this will verify that the clinical trial is conducted and data are generated, documented and reported in compliance with the protocol, GCP and the applicable regulatory requirements.

The Trial Management Group (TMG) will be comprised by the Chief Investigator together with the PC-CTU Senior Trial Manager, the senior co-investigators (Prof Jebb and Prof Aveyard), two PPI members, and one independent statistician (Dr. Mei-Man Lee). The TMG will be responsible for the monitoring of all aspects of the trial’s conduct and progress and will ensure that the protocol is adhered to and that appropriate action is taken to safeguard participants and the quality of the trial itself. The TMG will meet regularly throughout the course of the trial.

## Risk Assessment

The TMG will conduct a risk assessment and develop a trial specific monitoring plan before the first participant is recruited. The risk assessment will identify any trial specific risks (to both the participants’ safety and well-being and the integrity of the trial data).

Based on the outcome of this assessment, a trial specific monitoring plan will be developed to detail strategies to be employed to minimise the trial specific risks identified.

## Trial Steering Committee / Data Monitoring Committee

Due to the low risk nature of the PC-SHOP trial and that it is an open label trial, the Trial Management Group will act as the Trial Steering and Data Monitoring Committee to provide oversight of all matters relating to participant safety and data quality.

# ETHICAL AND REGULATORY CONSIDERATIONS

## Declaration of Helsinki

The Chief Investigator will ensure that this study is conducted in accordance with the principles of the Declaration of Helsinki 2013.

## Guidelines for Good Clinical Practice

The Chief Investigator will ensure that this study is conducted in accordance with relevant regulations and with Good Clinical Practice.

## Approvals

The protocol, informed consent form, participant information sheet and any other relevant intervention materials will be submitted to an appropriate Research Ethics Committee (REC), and host institution(s) for written approval.

The Chief Investigator will submit and, where necessary, obtain approval from the above parties for all substantial amendments to the original approved documents.

## Reporting

The CI shall submit once a year throughout the study, or on request, an Annual Progress report to the REC Committee, host organisation and Sponsor. In addition, an end-of-study notification and final report will be submitted to the same parties.

## Participant Confidentiality

Data will be kept in accordance with the Data Protection Act (DPA) 2018. We have discussed the data management and quality assurance of this study with a Senior Trials Manager from the UKCRC registered Primary Care Clinical Trials Unit (CTU). We agreed that they will provide general advice and support to ensure we follow CTU best practice but this feasibility study will not be formally adopted in the CTU portfolio. Participant identifiable information will be available to the person conducting follow ups as it is important that these data are known to them. Otherwise, confidentiality will be maintained and no-one outside the study team will have access to either the CRFs or the database.

Participants will only be identified on study documents by use of a unique study ID which cannot be used to identify individual participants. The central research team will store all CRFs and other study data documents, including the consent form, securely prior to data entry in a locked cabinet, in a locked room within a restricted access building with keypad access control system. CRFs and all other documents holding participant identifiable information will be anonymised as soon as possible with the process of management being outlined in detail within the ethics application and in the Data Management Plan.

Participant identifiable data will be required for the duration of the trial due to the intervention and trial procedures. Contact information will be destroyed at the end of the study. Participants will be identified in study documentation only by a unique identifier.

The database without contact details will be anonymised and a secure compact disc containing the link between identification number and participant identifiable information will be stored in a secure archiving facility.

The central research team will need to share the participant's name and clubcard number with the commercial partner (Tesco) so that the central research team can access the participant’s shopping data, which is needed to deliver the intervention. No other information will be shared with Tesco.

The trial management team will monitor confidentiality and consent will be sought from participants for the PC-SHOP research team to have direct access to participant medical records.

The study coordination centre will obtain approval from the Health Research Authority (HRA).

## Expenses and Benefits

As a result of participation in the PC-SHOP trial participants, will be asked to attend 2 or 3 study visits at their local GP practice. In order to minimise the burden on participants of claiming travel expenses (providing receipts, recording mileage, completing forms) the central research team will offer each participant a £10 gift card at the 3-month study visit.

For the qualitative sub-study, a £20 gift card will be provided by post after the interview has been completed.

# FINANCE AND INSURANCE

## Funding

This study will be funded by the National Institute for Health Research (NIHR) Collaboration for Leadership in Applied Health Research and Care Oxford at Oxford Health NHS Foundation Trust. The research funding will be administered by the administrative and finance teams within the Nuffield Department of Primary Care Health Sciences.

## Insurance

The University of Oxford will act as Sponsor for the PC-SHOP trial.

The University has a specialist insurance policy in place which would operate in the event of any participant suffering harm as a result of their involvement in the research (Newline Underwriting Management Ltd, at Lloyd’s of London).

# PUBLICATION POLICY

The investigators within the central research team will be involved in reviewing drafts of the manuscripts, abstracts, press releases and any other publications arising from the study. The main paper reporting the primary outcome will also include data on the secondary outcomes. Other qualitative outcomes, including acceptability of the intervention, may be reported in additional papers.

The trial results will be published and all who meet the criteria for authorship will be listed as authors. Authorship will be determined in accordance with the ICMJE guidelines and other contributors will be acknowledged.

We will submit the paper to NIHR for approval before submission.

# REFERENCES

1. Berneis KK, Krauss RM. Metabolic origins and clinical significance of LDL heterogeneity. J Lipid Res 2002;**43**(9):1363-79.

2. Mozaffarian D, Micha R, Wallace S. Effects on coronary heart disease of increasing polyunsaturated fat in place of saturated fat: a systematic review and meta-analysis of randomized controlled trials. PLoS Med 2010;**7**(3):e1000252.

3. Hooper L, Martin N, Abdelhamid A, et al. Reduction in saturated fat intake for cardiovascular disease. Cochrane Database Syst Rev 2015;**6**:CD011737.

4. Vafeiadou K, Weech M, Sharma V, et al. A review of the evidence for the effects of total dietary fat, saturated, monounsaturated and n-6 polyunsaturated fatty acids on vascular function, endothelial progenitor cells and microparticles. Br J Nutr 2012;**107**(3):303-24.

5. (NICE) NIoHaCE. Prevention of Cardiovascular disease. London 2010.

6. Vafeiadou K, Weech M, Altowaijri H, et al. Replacement of saturated with unsaturated fats had no impact on vascular function but beneficial effects on lipid biomarkers, E-selectin, and blood pressure: results from the randomized, controlled Dietary Intervention and VAScular function (DIVAS) study. Am J Clin Nutr 2015;**102**(1):40-8.

7. Reidlinger DP, Darzi J, Hall WL, et al. How effective are current dietary guidelines for cardiovascular disease prevention in healthy middle-aged and older men and women? A randomized controlled trial. The American journal of clinical nutrition 2015;**101**(5):922-30.

8. Rees K, Dyakova M, Wilson N, et al. Dietary advice for reducing cardiovascular risk. Cochrane Database Syst Rev 2013;**12**:CD002128.

9. Lin JS, O'Connor E, Whitlock EP, et al. Behavioral counseling to promote physical activity and a healthful diet to prevent cardiovascular disease in adults: a systematic review for the U.S. Preventive Services Task Force. Ann Intern Med 2010;**153**(11):736-50.

10. Kulick D, Langer RD, Ashley JM, et al. Live well: a practical and effective low-intensity dietary counseling intervention for use in primary care patients with dyslipidemia--a randomized controlled pilot trial. BMC Fam Pract 2013;**14**:59.

11. Neal B, Sacks G, Swinburn B, et al. Monitoring the levels of important nutrients in the food supply. Obes Rev 2013;**14 Suppl 1**:49-58.

12. Michie S, Abraham C, Whittington C, et al. Effective techniques in healthy eating and physical activity interventions: a meta-regression. Health Psychol 2009;**28**(6):690-701.

13. Atkins L, Michie S. Designing interventions to change eating behaviours. Proceedings of the Nutrition Society 2015;**74**(02):164-70.

14. Broekhuizen K, Kroeze W, van Poppel MN, et al. A systematic review of randomized controlled trials on the effectiveness of computer-tailored physical activity and dietary behavior promotion programs: an update. Ann Behav Med 2012;**44**(2):259-86.

15. Milliron BJ, Woolf K, Appelhans BM. A point-of-purchase intervention featuring in-person supermarket education affects healthful food purchases. J Nutr Educ Behav 2012;**44**(3):225-32.

16. Huang A, Barzi F, Huxley R, et al. The effects on saturated fat purchases of providing internet shoppers with purchase- specific dietary advice: a randomised trial. PLoS Clin Trials 2006;**1**(5):e22.

17. Michie S, van Stralen MM, West R. The behaviour change wheel: a new method for characterising and designing behaviour change interventions. Implement Sci 2011;**6**:42.

18. Foundation BH. Cut the Saturated Fat. BHF 2015.

19. Foundation BH. Eating Well. BHF 2012.

20. Greaves C, Gillison F, Stathi A, et al. Waste the waist: a pilot randomised controlled trial of a primary care based intervention to support lifestyle change in people with high cardiovascular risk. Int J Behav Nutr Phys Act 2015;**12**:1.

21. Pechey R, Jebb SA, Kelly MP, et al. Socioeconomic differences in purchases of more vs. less healthy foods and beverages: analysis of over 25,000 British households in 2010. Soc Sci Med 2013;**92**:22-6.

# APPENDIX A: STUDY FLOW CHART


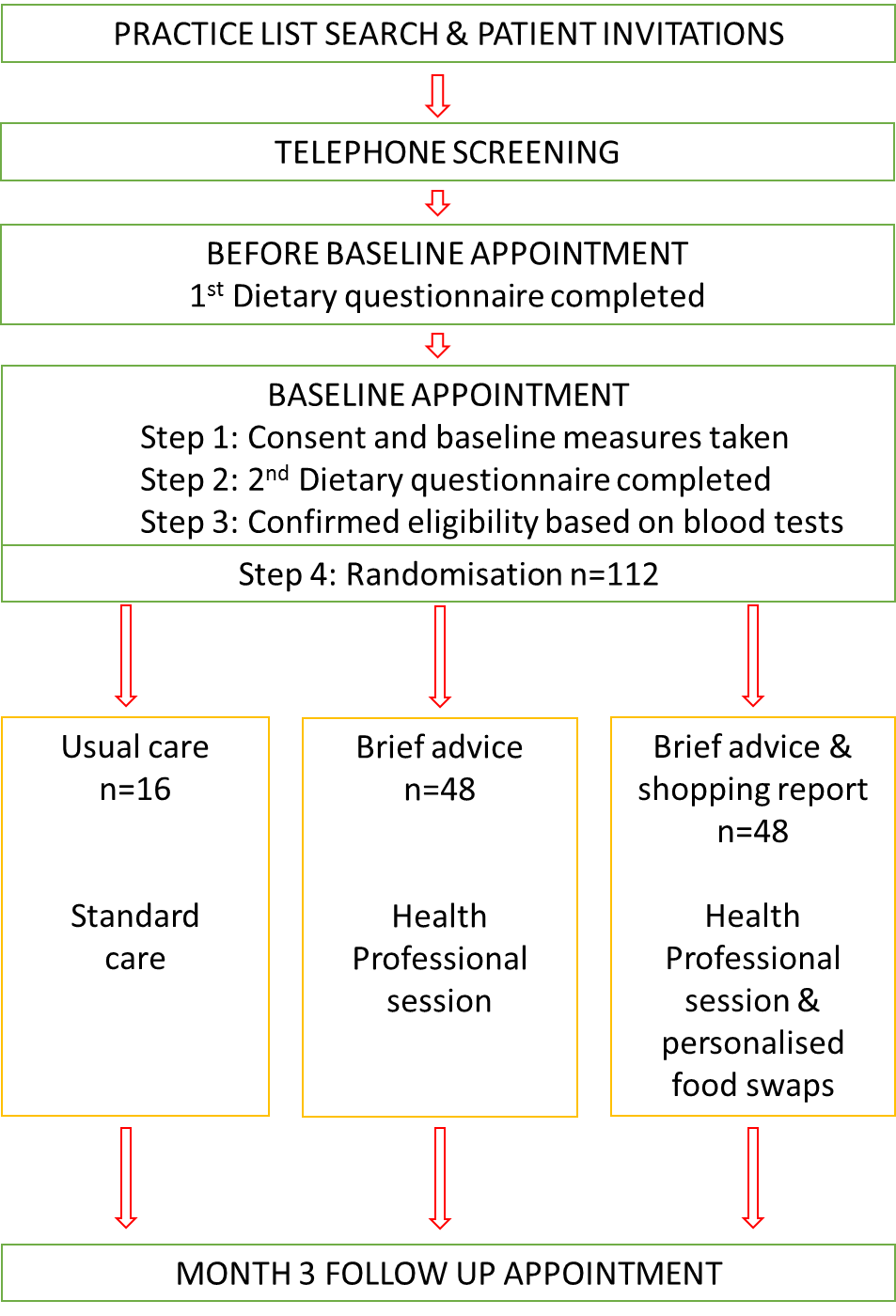


# APPENDIX B: SCHEDULE OF STUDY PROCEDURES

|  | **Baseline**  **visit 1** | **Baseline**  **visit 2** | **Month 3**  **visit** |
| --- | --- | --- | --- |
| **Length of visit** | 60 min | 15 min | 45 min |
| **Who conducts** | Central research team | Health  professional | Central research team |
| **Procedures** |  |  |  |
| Informed consent | √ |  |  |
| Eligibility assessment | √ |  |  |
| Randomisation | √ |  |  |
| Demographics/shopping behaviours | √ |  | √ |
| Medical history | √ |  | √ |
| Weight and height | √ |  | √ |
| Blood pressure | √ |  | √ |
| Blood sample | √ |  | √ |
| Medication review | √ |  | √ |
| 24h dietary recall | √ |  | √ |
| Tesco’s club card number | √ |  | √ |
| Questionnaires about the intervention | √ |  | √ |
| Intervention delivery |  | √ |  |

#

# APPENDIX C: QUALITATIVE SUB-STUDY TOPIC GUIDE

INTRODUCTION

“Hello, this is <<Researcher Name>> calling from the PC-SHOP study. Am I speaking with <<Participant Name>>?

You recently participated in our study and kindly agreed to tell us your experiences of trying to reduce the amount of saturated fat you were eating.

As a quick reminder, the study involved advice from the nurse at your GP surgery and some suggestions for healthier swaps based on the types of food you usually purchase. Does that sound familiar?

Some people have found having the British Heart Foundation leaflet and their shopping report in front of them helpful to jog your memory. If you have yours on hand, that would be great. But not a worry if not.

I’m going to ask roughly 10 questions about your experience of the study. These questions will help us cover everything and make sure that we do not overrun. This call should not last any longer than 30 minutes.

If you are agreeable, I will also record this call to allow us to analyse what you said in detail. Is it OK if I press record now?

NB: IF YES, RESEARCHER SHOULD PRESS RECORD HERE

NB: IF NO, RESEARCHER SHOULD THANK PARICIPANT AND END THE INTERVIEW NOW WITH AN EXPLANATION FOR THE REASONS WHY.

Please do give me your honest reflections about your experience, warts and all. We will not tell your GP what you say and we really want to hear about your experience to help us improve the support we give to people in the future.

Do you have any questions?

INFORMED CONSENT

OK. Before we begin, I have to ask you for your permission to have this call – something we call informed consent. To do this, I will read seven statements. Please reply by saying ‘yes’ at the end of each statement if you agree with it.

NB: RESEARCHER SHOULD INITIAL HARD COPY OF CONSENT FORM

| 1 | Do you confirm that you have read the Participant Information Sheet (version 5; dated 16thJanuary 2019), that you have had the opportunity to consider the information, ask questions and have had these questions answered satisfactorily? |
| --- | --- |
| 2 | Do you understand that your participation is voluntary and that you are free to withdraw at any time without having to give a reason, and without your medical care and legal rights being affected? |
| 3 | Do you understand that responsible members of the University of Oxford and the relevant NHS Trust(s) may be given access to the data collected during this call for monitoring and/or audit purposes to ensure that our research is complying with applicable regulations? |
| 4 | Do you consent to the interview being audio recorded? |
| 5 | Do you agree that anonymised quotes of your call may be included in reports that may be accessed freely by members of the public? It will not be possible for you to be identified in any of these reports. |
| 6 | Do you consent to take part in this study? |

NB: IF PARTICIPANT DOES NOT REPLY YES TO ALL STATEMENTS, RESEARCHER SHOULD THANK PARTICIPANT AND END THE INTERVIEW NOW. EXPLAIN THE REASONS WHY.

Great. I have signed a consent form on behalf of us both. You will receive a copy of the consent form in the post, along with your £20 store card as a thank you for your time.

How about I start by asking what prompted you to join the study?”

_________________________________________________________________________

QUESTIONS

| Intervention Components | Questions & Probes | Targeted Determinant | Intervention Function |
| --- | --- | --- | --- |
|  | What’s your understanding about the effects fat can have on your health?  Probes:  How do you think the fat in your food might affect your health?  How do you think about the cholesterol in your blood and its effects on your body? | Knowledge  Health Values | Education |
| BRIEF ADVICE SESSION DELIVERED BY A PRACTICE NURSE | | | |
| Discussion of CVD risk and motivational advice | What did you think about the advice you received from the nurse?  Probes:  What do you remember the nurse discussing about your recent blood results?  Did you feel there were important issues that weren’t discussed in this session? Give examples. | Knowledge  Health Values | Education  Persuasion |
|  | Did you try to make any changes to your diet after speaking with the nurse?  Probes:  In what ways did you think differently about your food when you went shopping?  How encouraged were you to start changing your diet?  If you didn’t change anything, what do you think is the reason for that? | Health Values | Education  Persuasion |
| BHF Leaflet | What did you think about the British Heart Foundation leaflet?  Probes:  How did it help your understanding about fat in your diet?  How motivated were you to act differently e.g. begin reading food labels, buying different foods? | Knowledge  Skills  Self-efficacy | Education  Persuasion  Training |
|  | Is there any other support your nurse or GP could provide, which you feel would help you to improve your diet?  Probes:  Did you get advice from anyone or anywhere else? If yes, how helpful was that advice? | - | - |
| PERSONALISED FEEDBACK ON FOOD SHOPPING | | | |
| Summary of % SFA in previous food purchases and top 3 foods contributing to SFA | What did you think about the shopping reports?  Probes:  How did you feel about us looking at your food shopping?  Did you find the feedback helpful? | Knowledge  Health Values  Behavioural regulation | Education  Persuasion |
| Suggested swaps | And what did you think about the healthier swaps suggested?  Probes:  Did you try the swaps?  Have you stuck to the swaps?  In what ways did you make any other swaps not suggested in the report?  Were the swaps reasonable in price, taste, appearance or brand prevent or encourage you to change your shopping? | Self-efficacy & behavioural regulation | Education  Training  Enablement |
| CONTEXUAL FACTORS & SUSTAINABILITY | | | |
|  | Was there anything else that influenced your food shopping?  Probes:  What other information would you like to have at hand in order to help you improve your food choices?  Do you feel anyone else in your household affected your shopping choices?  Is anyone else in your household using the swaps suggestions? | - | Contextual Factors and Sustainability |
| FUTURE INTERVENTIONS | | | |
|  | Imagine we ran this study again. How would you feel about health advice coming from someone else other than your GP, for example a supermarket pharmacist? | - | Who and where behaviour is influenced |
|  | Lastly, are there any final thoughts or experiences about the study that you would like to share?  Probe  Are there any bits that you felt worked or really didn’t work?  Do you have any other suggestions on how we could improve this study? | - | - |

“Well that’s the end of this call.

The intention was to discuss your experiences of the PC-SHOP study. To summarise, we spoke about your understanding of saturated fat and its effect on your health, your thoughts on the advice and feedback you received, and some recommendations for future research.

You will shortly receive a £20 store card in the mail, as well as copy of the researcher-initialed consent form.

If you have any questions about this research, please do not hesitate to contact a member of the research team.

Thank you very much for your time. I’ve really enjoyed hearing about your experiences and I hope you’ve enjoyed sharing them with me today. Thank you very much for taking part in this study. Goodbye”.

___________________________________________________________________________

END

# APPENDIX C: AMENDMENT HISTORY

| **Amendment No.** | **Protocol Version No.** | **Date issued** | **Author(s) of changes** | **Details of Changes made** |
| --- | --- | --- | --- | --- |
| 1 | 2.0 | 1^st^ October 2017 | Carmen M Piernas Sanchez | - Added details of data sharing with Tesco to the introduction.  - Changed to fingerprick blood analyses, samples taken by study team.  - Added a new secondary outcome: intake of high SFA foods as it is important to know changes in those in addition to changes in total SFA intake  - Changed slightly the study procedures to clarify that interested participants should contact the study team via email and to explain that we will ask participants to provide a dietary recall before the baseline visit. These changes to the procedures will improve efficiency of the study.  - Changes/clarifications to inclusion/exclusion criteria. One new exclusion criterion is those who started medication in the last 3 months.  - Added clarification about study consent with regards to Tesco’s information  - Only changes to intervention acceptability will be collected so no changes in demographics, health behaviours or shopping habits will be collected at follow up as baseline information is sufficient.  - Added a paragraph about collecting till receipts as a contingency plan in case the collaborating supermarket is unable to provide the data needed to generate the feedback report.  - Paper CRFs will only be needed when computer/internet not working  - Deleted some information from the purchasing report which won’t be provided (total kcal)  - Clarify that SAEs for this study will be considered unexpected  - Added details of how the dietary data is collected and stored |
| 2 | 3.0 | 1^st^ February 2018 | Carmen M Piernas Sanchez | - Added additional funding source  - Few clarifications/typos throughout  - Clarification in inclusion criteria that participants need to have a registered clubcard exclusively registered under their name  - Clarification to exclusion criteria so that people recently involved in other studies will not be eligible if the nature of the intervention in which they participated will likely affect the outcomes of this study.  - Use of RedCap for CRF data collection  - Randomisation will not be stratified by GP and block size will be 7, as suggested by the statistician to avoid problems with block sizes.  - More information about our primary analysis as it wasn’t clear in the previous paragraph. |
| 3 | 4.0 | 11^th^ December 2018 | Carmen M Piernas Sanchez | - Updated details of Trial Team  - Letter with blood tests to be sent at the end of the study to participants  - Added details of the qualitative sub-study and topic guide in the Appendix section  - Added more details of other lipids to be measured as part of the study: non-HDL cholesterol and total/HDL cholesterol ratio |
