# Supplementary material for: Evaluation of an intervention to provide brief support and personalized feedback on food shopping to reduce saturated fat intake (PC-SHOP): A randomized controlled trial
Source: PLoS Med. 2020 Nov 5;17(11):e1003385. doi: 10.1371/journal.pmed.1003385 (PMC7643942; doi:10.1371/journal.pmed.1003385)
Supplement: S2 Appendix — (DOCX) [file pmed.1003385.s002.docx]

## List of inclusion and exclusion criteria

Inclusion criteria

- Aged ≥18 years
- LDL cholesterol ≥ 3 mmol/L as measured during recruitment
- Willing to make changes to their diet in order to reduce CVD risk
- Have responsibility for the majority of the household food/grocery shopping (complete at least half of their household shopping)
- Shopping at the collaborating grocery store (at least every 2 weeks in store and/or online)
- Have a loyalty card registered exclusively under their name before recruitment
- Access and ability to use a computer with internet connection
- Willing and able to give informed consent for participation in the study

Exclusion criteria

- Unable to read and understand the instructions provided in English
- Self-reported pregnancy, or planning to become pregnant during the course of the study
- Started cholesterol-lowering medication (e.g. statins) in the last 3 months
- Planned changes to cholesterol-lowering medication in the next 3 months
- Existing cardiovascular conditions: heart attack, stroke, or new diagnosis of atrial fibrillation within the last 3 months; heart failure of grade I New York Heart Association, and more severe or prolonged QT syndrome, angina, arrhythmia, or familial hyperlipidaemia
- Currently or recently (within the last 3 months) participating in another intervention study which likely affects the outcomes measured in this study
- Participants GP judges them unsuitable for the study
