# Supplementary material for: Evaluation of an intervention to provide brief support and personalized feedback on food shopping to reduce saturated fat intake (PC-SHOP): A randomized controlled trial
Source: PLoS Med. 2020 Nov 5;17(11):e1003385. doi: 10.1371/journal.pmed.1003385 (PMC7643942; doi:10.1371/journal.pmed.1003385)
Supplement: S3 Appendix — (DOCX) [file pmed.1003385.s003.docx]

## Qualitative questionnaire

Example of questionnaires used to assess changes in knowledge, motivation and acceptability of the intervention

**A. Knowledge & Information**

1. Do you think saturated fat (i.e. the type of fat found in animal products like meat or butter) affects cardiovascular health?
   1. Yes
   2. No
   3. Don’t know

**B. Action & Motivation**

1. Have you tried to reduce the amount of saturated fat you eat in the last 3 months?
   1. Yes
   2. No
   3. Don’t know
2. If yes, why are you trying to reduce the amount of saturated fat you eat?
   1. Reduce the risk of heart attack or stroke
   2. Avoid long-term use of lipid medication (e.g. statins)
   3. Other reasons: ______________________________

**C. Confidence**

1. How confident are you that you know the major sources of saturated fat in your diet?
   1. Very confident
   2. Somewhat confident
   3. Not very confident
2. Do you think you would be able to reduce the amount of saturated fat you eat over the next few months?
   1. Yes
   2. No
   3. Don’t know

**D. Expectations**

1. **What were you hoping to achieve by participating in this study?**
   1. **To be able to identify healthier/less healthy foods in my diet**
   2. **To avoid the need to take tablets to improve my health**
   3. **To reduce my risk of cardiovascular disease over the next few years**
   4. **Other reasons: _________________________________**

**F. Intervention-specific questions (Brief advice session + shopping report group)**

1. **On a scale of 1-5, how helpful was the British Heart Foundation booklet to reduce the amount of saturated fat you eat?**

**Very unhelpful 1 2 3 4 5 Very helpful**

1. **On a scale of 1-5, how helpful was the advice session led by your health care provider to reduce the amount of saturated fat you eat?**

**Very unhelpful 1 2 3 4 5 Very helpful**

1. **Thinking about the advice you received from your health care provider, which aspects of that did you find:**

**Helpful (tick all that apply):**

1. **Motivated me to try to reduce my blood cholesterol**
2. **Motivated me to shop for products with lower saturated fat**
3. **Helped me change my diet to consume less saturated fat**
4. **Helped me to understand more about the importance of diet for my health**
5. **Helped me to understand the importance of maintaining a healthy lifestyle**
6. **Other: please explain_________________________________________________**

**Unhelpful: (tick all that apply)**

1. **I didn’t understand the content of the session**
2. **The session was too short and basic**
3. **It was too general to motivate me to change my diet**
4. **I didn’t think it was relevant to me**
5. **Other: please explain_________________________________________________**
6. **On a scale of 1-5, how helpful was your shopping report to reduce the amount of saturated fat on the food you purchased?**

**Very unhelpful 1 2 3 4 5 Very helpful**

1. **Did you use the shopping report to help you buy foods with lower saturated fat content in your next shopping trips?**
   1. **Always**
   2. **Most of the time**
   3. **Occasionally**
   4. **Never**
2. **Thinking about the advice and the food options offered in your monthly shopping report, which aspects of that did you find (drop down menu):**

**Helpful (tick all that apply):**

- 1. **Provided a wide range of swaps to help me reduce saturated fat in my shopping**
  2. **Provided good alternatives to the products I usually buy**
  3. **It was clear and informative**
  4. **Motivated me to try to reduce my blood cholesterol**
  5. **Motivated me to shop products with lower saturated fat**
  6. **Helped me change my diet even when eating out at a restaurant**
  7. **Other: please explain _____________________________________________________**

**Unhelpful: (tick all that apply)**

- 1. **Forgot to bring the report to my next shop**
  2. **Forgot about the options offered**
  3. **I didn’t understand the content of the report**
  4. **The report missed important information e.g. price**
  5. **The options offered were not available at the store**
  6. **The options offered were more expensive**
  7. **I didn’t like most of the options offered**
  8. **My family did not accept the options offered**
  9. **Other: please explain ________________________________________________________**

1. **What else would be helpful for you in order to improve your diet?**

**Please include as much detail as you like:**

**____________________________________________________________________________________________________________________________________________________________________________________________________________________________________________________________________________________________________**

1. **Please include any other comments you may have with regards to this study:**

**____________________________________________________________________________________________________________________________________________________________________________________________________________________________________________________________________________________________________**
