# Supplementary material for: Evaluation of an intervention to provide brief support and personalized feedback on food shopping to reduce saturated fat intake (PC-SHOP): A randomized controlled trial
Source: PLoS Med. 2020 Nov 5;17(11):e1003385. doi: 10.1371/journal.pmed.1003385 (PMC7643942; doi:10.1371/journal.pmed.1003385)
Supplement: S4 Appendix — (DOCX) [file pmed.1003385.s004.docx]

## Supplementary tables

## Table A. Mean dietary intakes (standard deviation) estimated using 2 x 24h dietary recalls.

|  | **Control (n=16)**  **Mean (SD)** | | | | **Brief Support (n=46)**  **Mean (SD)** | | | | **Brief Support plus Shopping Advice (n=44)**  **Mean (SD)** | | | |
| --- | --- | --- | --- | --- | --- | --- | --- | --- | --- | --- | --- | --- |
|  | **Baseline** | | **Follow up** | | **Baseline** | | **Follow up** | | **Baseline** | | **Follow up** | |
| Saturated fat (% EI) | 12.3 | 3.3 | 12.3 | 4.3 | 12.9 | 3.6 | 12.1 | 3.3 | 13.7 | 3.4 | 12.8 | 3.2 |
| Saturated fat (kcal) | 233 | 76 | 242 | 124 | 261 | 127 | 223 | 120 | 260 | 102 | 231 | 89 |
| Total fat (% EI) | 34.0 | 8.1 | 31.0 | 8.3 | 33.7 | 7.1 | 31.4 | 6.7 | 36.1 | 8.1 | 34.2 | 8.2 |
| Total fat (kcal) | 649 | 203 | 623 | 284 | 685 | 310 | 575 | 281 | 693 | 269 | 614 | 243 |
| Cakes/biscuits/desserts (% EI) | 9.7 | 6.3 | 12.1 | 11.1 | 9.8 | 9.0 | 7.2 | 7.8 | 9.3 | 7.1 | 9.8 | 9.4 |
| Meat (% EI) | 4.6 | 4.5 | 5.2 | 6.7 | 7.7 | 6.0 | 5.4 | 6.5 | 4.9 | 5.5 | 4.9 | 5.5 |
| Poultry (%EI) | 3.8 | 3.8 | 3.6 | 4.6 | 2.6 | 3.8 | 4.3 | 6.5 | 3.2 | 4.1 | 5.1 | 7.4 |
| Cheese (High fat, %EI) | 1.3 | 1.7 | 1.4 | 1.9 | 2.4 | 2.9 | 1.6 | 2.4 | 3.1 | 3.0 | 1.8 | 3.0 |
| Cheese (Low fat, %EI) | 0.5 | 1.0 | 0.0 | 0.1 | 0.4 | 1.3 | 0.5 | 1.5 | 0.1 | 0.5 | 0.8 | 1.6 |
| Yoghurt (High fat, %EI) | 0.5 | 1.5 | 0.0 | 0.0 | 0.5 | 1.3 | 0.7 | 2.2 | 1.2 | 2.7 | 0.4 | 1.5 |
| Yoghurt (Low fat, %EI) | 1.1 | 1.8 | 1.4 | 2.7 | 0.9 | 1.7 | 1.8 | 2.7 | 1.1 | 2.1 | 1.7 | 2.4 |
| Spread (High fat, %EI) | 2.9 | 2.8 | 1.0 | 1.8 | 1.7 | 2.8 | 0.8 | 2.3 | 2.2 | 3.1 | 1.0 | 2.1 |
| Spread (Low fat, %EI) | 0.6 | 1.1 | 0.7 | 1.6 | 0.5 | 1.1 | 0.8 | 2.0 | 0.7 | 1.4 | 0.7 | 1.1 |
| Salty snacks (%EI) | 1.5 | 2.7 | 1.3 | 1.9 | 2.2 | 3.8 | 3.0 | 5.8 | 1.9 | 3.9 | 1.4 | 4.0 |
| Polyunsaturated fat (% EI) | 6.5 | 3.0 | 5.4 | 1.7 | 6.2 | 2.4 | 5.5 | 1.9 | 6.5 | 2.6 | 6.6 | 2.8 |
| Polyunsaturated fat (kcal) | 125 | 66 | 114 | 68 | 128 | 75 | 101 | 52 | 128 | 69 | 121 | 68 |
| Monounsaturated fat (% EI) | 15.1 | 4.4 | 13.3 | 3.5 | 14.6 | 3.3 | 13.8 | 3.3 | 15.9 | 4.3 | 14.8 | 4.9 |
| Monounsaturated fat (kcal) | 291 | 101 | 267 | 114 | 296 | 134 | 251 | 123 | 305 | 127 | 262 | 110 |
| Total energy intake (kcal) | 1933 | 495 | 1934 | 577 | 1986 | 638 | 1809 | 738 | 1867 | 502 | 1761 | 546 |
| Total sugars (% EI) | 23.5 | 7.6 | 26.7 | 8.6 | 23.4 | 7.4 | 24.3 | 9.6 | 21.5 | 7.7 | 23.4 | 7.3 |
| Total sugars (kcal) | 454 | 179 | 497 | 176 | 460 | 224 | 438 | 251 | 378 | 134 | 399 | 162 |
| Fibre (g/1000 kcal) | 8.5 | 2.7 | 8.4 | 2.8 | 8.8 | 3.7 | 8.8 | 3.3 | 8.7 | 3.1 | 9.1 | 3.3 |
| *EI = energy intake | | | | | | | | | | | | |

## Table B. Changes in dietary intakes estimated using 2 x 24h dietary recalls: primary, secondary and exploratory outcome results by group allocation.

|  | **Change from baseline within group** | | | | | | | | | **Between-group differences adjusted for baseline and practice*** | | | | | | | | | | | | |
| --- | --- | --- | --- | --- | --- | --- | --- | --- | --- | --- | --- | --- | --- | --- | --- | --- | --- | --- | --- | --- | --- | --- |
|  | **Control** | | | **Brief Support**  **(BS)**  **(n=46)** | | | **Brief Support plus**  **Shopping Feedback (SF)**  **(n=44)** | | | **BS vs control** | | | | **SF vs control** | | | | **SF vs BS** | | | | |
| **Primary Outcome** | Mean | 95% CI | | Mean | 95% CI | | Mean | 95% CI | | Mean | 95% CI | | P value | Mean | 95% CI | | P value | Mean | 95% CI | | P value | |
| **Saturated fat (% EI)** | -0.1 | (-1.8, | 1.7) | -0.7 | (-1.8, | 0.3) | -0.9 | (-2.0, | 0.2) | -0.3 | (-2.1, | 1.4) | 0.709 | -0.1 | (-1.9, | 1.7) | 0.901 | 0.2 | (-1.1, | 1.5) | 0.736 | |
| **Secondary Outcomes** |  |  |  |  |  |  |  |  |  |  |  |  |  |  |  |  |  |  |  |  |  | |
| **Saturated fat (kcal)** | 8.8 | (-58.3, | 76.0) | -38.4 | (-78.0, | 1.2) | -29.6 | (-70.2, | 10.9) | -25.3 | (-87.1, | 36.5) | 0.419 | -15.8 | (-78.2, | 46.6) | 0.616 | 9.5 | (-35.1, | 54.0) | 0.674 | |
| **Total fat (% EI)** | -3 | (-7.6, | 1.7) | -2.3 | (-5.1, | 0.4) | -1.9 | (-4.7, | 0.9) | 0.4 | (-3.9, | 4.6) | 0.868 | 2.7 | (-1.6, | 7.1) | 0.217 | 2.4 | (-0.8, | 5.5) | 0.138 | |
| **Total fat (kcal)** | -25.9 | (-202.6, | 150.9) | -109.8 | (-214.0, | -5.6) | -79.4 | (-186.0, | 27.2) | -56.4 | (-208.0, | 95.1) | 0.462 | -11.4 | (-164.5, | 141.7) | 0.883 | 45 | (-64.7, | 154.8) | 0.418 | |
| **Cakes/biscuits/desserts (% EI)** | 2.4 | (-2.8, | 7.6) | -2.6 | (-5.7, | 0.4) | 0.5 | (-2.7, | 3.6) | -4.9 | (-10.0, | 0.3) | 0.064 | -2.3 | (-7.6, | 2.9) | 0.375 | 2.5 | (-1.2, | 6.3) | 0.182 | |
| **Meat (% EI)** | 0.6 | (-3.0, | 4.2) | -2.3 | (-4.5, | -0.2) | 0 | (-2.2, | 2.2) | -0.5 | (-4.1, | 3.1) | 0.791 | -0.3 | (-3.9, | 3.3) | 0.86 | 0.2 | (-2.5, | 2.8) | 0.902 | |
| **Poultry (% EI)** | -0.2 | (-4.1, | 3.7) | 1.6 | (-0.7, | 3.9) | 1.9 | (-0.5, | 4.2) | 0.6 | (-3.3, | 4.5) | 0.751 | 1.7 | (-2.2, | 5.6) | 0.38 | 1.1 | (-1.7, | 3.9) | 0.433 | |
| **Higher fat cheese (% EI)** | 0.2 | (-1.6, | 1.9) | -0.9 | (-1.9, | 0.2) | -1.3 | (-2.3, | -0.2) | -0.1 | (-1.6, | 1.5) | 0.928 | 0 | (-1.5, | 1.6) | 0.968 | 0.1 | (-1.0, | 1.2) | 0.854 | |
| **Lower fat cheese (% EI)** | -0.5 | (-1.3, | 0.3) | 0.2 | (-0.3, | 0.7) | 0.7 | (0.2, | 1.2) | 0.5 | (-0.3, | 1.3) | 0.227 | 0.8 | (0.0, | 1.7) | 0.053 | 0.3 | (-0.3, | 0.9) | 0.291 | |
| **Higher fat yoghurt**  **(% EI)** | -0.5 | (-1.6, | 0.5) | 0.2 | (-0.4, | 0.8) | -0.8 | (-1.4, | -0.2) | 0.7 | (-0.3, | 1.6) | 0.163 | 0.1 | (-0.8, | 1.1) | 0.781 | -0.5 | (-1.2, | 0.2) | 0.129 | |
| **Lower fat yoghurt (% EI)** | 0.3 | (-1.0, | 1.6) | 1 | (0.2, | 1.7) | 0.6 | (-0.2, | 1.3) | 0.8 | (-0.6, | 2.1) | 0.260 | 0.5 | (-0.9, | 1.9) | 0.468 | -0.3 | (-1.3, | 0.7) | 0.585 | |
| **Higher fat spread (% EI)** | -1.9 | (-3.5, | -0.3) | -0.8 | (-1.8, | 0.1) | -1.3 | (-2.2, | -0.3) | 0.1 | (-1.2, | 1.3) | 0.928 | 0 | (-1.2, | 1.3) | 0.975 | 0 | (-0.9, | 0.8) | 0.935 | |
| **Lower fat spread (% EI)** | 0.1 | (-0.7, | 0.9) | 0.3 | (-0.1, | 0.8) | 0 | (-0.5, | 0.5) | 0 | (-0.8, | 0.8) | 0.996 | -0.2 | (-1.0, | 0.7) | 0.669 | -0.2 | (-0.8, | 0.4) | 0.548 | |
| **Salty snacks (% EI)** | -0.2 | (-2.6, | 2.1) | 0.8 | (-0.6, | 2.2) | -0.5 | (-1.9, | 0.9) | 1.3 | (-1.3, | 3.8) | 0.327 | -0.3 | (-2.8, | 2.3) | 0.82 | -1.5 | (-3.4, | 0.3) | 0.097 | |
| **Exploratory Outcomes** |  |  |  |  |  |  |  |  |  |  |  |  |  |  |  |  |  |  |  |  |  | |
| **Polyunsaturated fat**  **(% EI)** | -1.1 | (-2.6, | 0.5) | -0.7 | (-1.6, | 0.2) | 0.2 | (-0.8, | 1.1) | 0 | (-1.3, | 1.3) | 0.969 | 1.2 | (-0.1, | 2.5) | 0.076 | 1.2 | (0.2, | 2.1) | 0.016 | |
| **Polyunsaturated fat (kcal)** | -11.2 | (-56.0, | 33.6) | -26.7 | (-53.2, | -0.3) | -6.9 | (-34.0, | 20.1) | -15.3 | (-50.8, | 20.2) | 0.395 | 6.8 | (-29.1, | 42.6) | 0.709 | 22.1 | (-3.7, | 47.8) | 0.093 | |
| **Monounsaturated fat (% EI)** | -1.8 | (-4.3, | 0.8) | -0.9 | (-2.4, | 0.6) | -1.1 | (-2.6, | 0.4) | 0.5 | (-1.8, | 2.8) | 0.681 | 1.3 | (-1.0, | 3.7) | 0.259 | 0.9 | (-0.8, | 2.6) | 0.317 | |
| **Monounsaturated fat (kcal)** | -23.5 | (-101.1, | 54.1) | -44.6 | (-90.4, | 1.1) | -42.8 | (-89.6, | 4.0) | -18.6 | (-84.4, | 47.1) | 0.575 | -5.6 | (-72.0, | 60.8) | 0.867 | 13 | (-34.7, | 60.7) | 0.589 | |
| **Non-efficacy Outcomes** |  |  |  |  |  |  |  |  |  |  |  |  |  |  |  |  |  |  |  |  |  | |
| **Total energy intake (kcal)** | 0.8 | (-365.7, | 367.4) | -176.9 | (-393.1, | 39.2) | -106.2 | (-327.2, | 114.9) | -152 | (-512.5, | 208.5) | 0.405 | -152.4 | (-515.8, | 211.0) | 0.407 | -0.4 | (-262.7, | 262.0) | 0.998 | |
| **Total sugars (% EI)** | 3.2 | (-0.7, | 7.1) | 0.9 | (-1.4, | 3.2) | 1.8 | (-0.5, | 4.2) | -1.7 | (-5.8, | 2.3) | 0.393 | -1.9 | (-6.0, | 2.1) | 0.349 | -0.2 | (-3.1, | 2.7) | 0.896 | |
| **Total sugars (kcal)** | 42.6 | (-49.8, | 135.0) | -21.5 | (-76.0, | 33.0) | 21.2 | (-34.6, | 76.9) | -60 | (-161.9, | 41.9) | 0.246 | -51.9 | (-155.6, | 51.8) | 0.323 | 8.1 | (-67.4, | 83.6) | 0.832 | |
| **Fibre (g/1000 kcal)** | -0.1 | (-1.6, | 1.3) | 0.1 | (-0.8, | 0.9) | 0.3 | (-0.5, | 1.2) | 0.2 | (-1.3, | 1.7) | 0.79 | 0.4 | (-1.1, | 1.9) | 0.592 | 0.2 | (-0.9, | 1.3) | 0.706 | |
| *** Estimates from linear regression models adjusting for GP practice and baseline, all p values >0.05; ** EI = energy intake** | | | | | | | | | | | | | | | | | | | | | |  |

## Table C. Mean nutrient and food group purchases (standard deviation) estimated from loyalty card data.

|  | **Control (n=15)**  **Mean (SD)** | | | | **Brief Support (n=44)**  **Mean (SD)** | | | | **Brief Support plus Shopping Advice (n=44)**  **Mean (SD)** | | | |
| --- | --- | --- | --- | --- | --- | --- | --- | --- | --- | --- | --- | --- |
|  | **Baseline** | | **Follow up** | | **Baseline** | | **Follow up** | | **Baseline** | | **Follow up** | |
| Saturated fat (% of TE) | 14.2 | 3.3 | 13.7 | 4.1 | 14.2 | 4.8 | 12.9 | 4.4 | 15.0 | 4.0 | 13.5 | 3.0 |
| Saturated fat (kcal per £ spent) | 63 | 19 | 59 | 28 | 59 | 21 | 53 | 21 | 61 | 19 | 54 | 18 |
| Total fat (% TE) | 38.3 | 6.6 | 38.3 | 7.5 | 36.1 | 10.2 | 34.8 | 9.5 | 36.2 | 8.2 | 35.9 | 6.6 |
| Total fat (kcal per £ spent) | 170 | 43 | 159 | 61 | 153 | 60 | 145 | 54 | 148 | 46 | 144 | 41 |
| Purchases of ALL products with >1.5% SFA (%TE) | 53.8 | 9.0 | 51.1 | 8.8 | 49.9 | 15.4 | 46.8 | 12.5 | 53.3 | 13.2 | 49.7 | 11.2 |
| Cakes/biscuits/desserts (%TE) | 13.3 | 10.8 | 11.6 | 8.0 | 13.0 | 9.8 | 10.3 | 8.6 | 15.8 | 15.2 | 12.8 | 10.2 |
| Meat (%TE) | 8.0 | 5.4 | 7.1 | 6.7 | 7.4 | 13.7 | 5.3 | 4.6 | 6.4 | 4.3 | 6.2 | 4.9 |
| Poultry (%TE) | 3.2 | 2.4 | 2.7 | 2.5 | 2.2 | 2.5 | 2.9 | 2.6 | 2.8 | 2.1 | 4.6 | 4.5 |
| Cheese (High fat, %TE) | 4.0 | 4.0 | 3.9 | 4.6 | 3.5 | 3.0 | 3.1 | 2.8 | 3.5 | 3.1 | 3.0 | 2.6 |
| Cheese (Low fat, %TE) | 0.1 | 0.2 | 0.2 | 0.3 | 0.1 | 0.2 | 0.3 | 0.5 | 0.4 | 0.6 | 0.5 | 0.9 |
| Milk/other diary (%TE) | 7.4 | 5.3 | 8.5 | 5.5 | 6.2 | 4.0 | 7.1 | 7.2 | 7.5 | 6.4 | 7.6 | 4.9 |
| Yoghurt (High fat, %TE) | 0.8 | 1.2 | 0.6 | 0.6 | 1.3 | 1.5 | 1.5 | 1.9 | 1.4 | 2.0 | 1.4 | 1.7 |
| Yoghurt (Low fat, %TE) | 0.1 | 0.3 | 0.1 | 0.4 | 0.4 | 0.6 | 0.6 | 0.9 | 0.3 | 0.5 | 0.7 | 0.9 |
| Spread (High fat, %TE) | 5.3 | 3.5 | 4.7 | 4.4 | 5.5 | 5.3 | 5.3 | 5.0 | 4.5 | 4.4 | 3.8 | 4.3 |
| Spread (Low fat, %TE) | 0.1 | 0.4 | 0.1 | 0.2 | 0.4 | 1.5 | 0.9 | 2.4 | 0.2 | 0.6 | 0.6 | 0.9 |
| Salty snacks (%TE) | 2.4 | 1.8 | 2.7 | 1.9 | 2.9 | 4.2 | 2.3 | 2.3 | 2.2 | 2.2 | 2.2 | 2.6 |
| Ready meals (High SFA, %TE) | 4.4 | 5.1 | 4.0 | 2.7 | 2.7 | 2.8 | 2.7 | 3.4 | 4.0 | 2.7 | 3.3 | 4.0 |
| Ready meals (Low SFA, %TE) | 0.7 | 0.7 | 1.4 | 1.5 | 1.2 | 1.4 | 1.0 | 1.1 | 1.3 | 1.3 | 1.3 | 1.3 |
| Energy density (kcal/g purchased) | 1.5 | 0.2 | 1.3 | 0.3 | 1.4 | 0.3 | 1.3 | 0.3 | 1.3 | 0.3 | 1.3 | 0.3 |
| Total sugars (% TE) | 22.5 | 7.5 | 20.2 | 8.0 | 22.8 | 8.9 | 23.0 | 7.8 | 22.8 | 10.5 | 22.7 | 7.6 |
| Total sugars (kcal per £ spent) | 95.2 | 22.5 | 86.9 | 37.4 | 96.2 | 40.2 | 92.1 | 34.0 | 92.3 | 40.6 | 90.8 | 33.5 |
| Fibre (g/1000 kcal purchased) | 9.9 | 1.8 | 9.2 | 3.4 | 10.2 | 3.0 | 11.5 | 4.2 | 12.2 | 4.2 | 13.6 | 9.0 |
| Salt (g/100g purchased) | 1.1 | 1.2 | 0.8 | 0.6 | 0.7 | 0.5 | 0.6 | 0.4 | 0.9 | 0.7 | 0.7 | 0.4 |
| Absolute basket cost (£ per week) | 32.0 | 23.4 | 30.5 | 19.6 | 35.7 | 38.9 | 36.9 | 23.5 | 33.1 | 24.1 | 35.6 | 23.3 |
| Relative basket cost (£ per Kg) | 3.4 | 0.6 | 3.4 | 1.0 | 3.5 | 1.3 | 3.2 | 0.8 | 3.3 | 0.9 | 3.4 | 0.9 |
| *TE = total energy in purchases | | | | | | | | | | | | |

## Table D. Changes in shopping behaviours estimated from loyalty card data: secondary and exploratory outcomes by group allocation.

|  | **Change from baseline within group** | | | | | | | | | **Between-group differences adjusted for baseline and practice*** | | | | | | | | | | | |
| --- | --- | --- | --- | --- | --- | --- | --- | --- | --- | --- | --- | --- | --- | --- | --- | --- | --- | --- | --- | --- | --- |
|  | **Control**    **(n=15)** | | | **Brief Support**  **(BS)**  **(n=44)** | | | **Brief Support plus**  **Shopping Feedback (SF)**  **(n=44)** | | | **BS vs control** | | | | **SF vs control** | | | | **SF vs BS** | | | |
| **Secondary Outcomes** | Mean | 95% CI | | Mean | 95% CI | | Mean | 95% CI | | Mean | 95% CI | | P value | Mean | 95% CI | | P value | Mean | 95% CI | | P value |
| **Saturated fat (% TE)** | -0.5 | (-2.3, | 1.2) | -1.3 | (-2.3, | -0.3) | -1.5 | (-2.5, | -0.5) | -0.8 | (-2.5, | 0.9) | 0.379 | -0.7 | (-2.4, | 1.0) | 0.411 | 0 | (-1.2, | 1.3) | 0.94 |
| **Saturated fat (kcal per £)** | -4.3 | (-13.5, | 4.8) | -6.5 | (-11.8, | -1.1) | -6.7 | (-12.0, | -1.3) | -4 | (-13.8, | 5.8) | 0.417 | -3.9 | (-13.7, | 5.9) | 0.429 | 0.1 | (-6.9, | 7.1) | 0.980 |
| **Total fat (% TE)** | 0 | (-3.9, | 3.9) | -1.2 | (-3.5, | 1.0) | -0.3 | (-2.6, | 2.0) | -2.3 | (-6.2, | 1.6) | 0.238 | -1.5 | (-5.4, | 2.4) | 0.436 | 0.8 | (-2.0, | 3.5) | 0.572 |
| **Total fat (kcal per £)** | -10.9 | (-35.7, | 13.8) | -8.8 | (-23.2, | 5.7) | -4.5 | (-19.0, | 9.9) | -8.2 | (-33.2, | 16.8) | 0.517 | -7.1 | (-32.3, | 18.1) | 0.579 | 1.1 | (-16.6, | 18.8) | 0.901 |
| **Food products >1.5% SFA (%TE)** | -2.7 | (-8.5, | 3.2) | -3.1 | (-6.6, | 0.4) | -3.6 | (-7.1, | -0.1) | -2.1 | (-7.8, | 3.5) | 0.452 | -1 | (-6.6, | 4.6) | 0.719 | 1.1 | (-2.9, | 5.2) | 0.584 |
| **Cakes/biscuits/desserts (% TE)** | -1.7 | (-7.6, | 4.1) | -2.7 | (-6.1, | 0.7) | -3 | (-6.4, | 0.4) | -0.9 | (-5.7, | 4.0) | 0.729 | 0.5 | (-4.4, | 5.5) | 0.826 | 1.4 | (-2.1, | 4.9) | 0.430 |
| **Meat (% TE)** | -0.8 | (-4.7, | 3.1) | -2.1 | (-4.4, | 0.2) | -0.2 | (-2.4, | 2.1) | -1.5 | (-3.9, | 0.8) | 0.200 | -0.4 | (-2.8, | 1.9) | 0.707 | 1.1 | (-0.6, | 2.7) | 0.204 |
| **Poultry (% TE)** | -0.5 | (-2.5, | 1.4) | 0.7 | (-0.4, | 1.9) | 1.8 | (0.6, | 2.9) | 0.7 | (-1.4, | 2.7) | 0.529 | 2 | (0.0, | 4.1) | 0.055 | 1.4 | (-0.1, | 2.9) | 0.072 |
| **Higher fat cheese (% TE)** | -0.1 | (-1.7, | 1.5) | -0.4 | (-1.3, | 0.5) | -0.5 | (-1.4, | 0.5) | -0.6 | (-2.2, | 1.0) | 0.457 | -0.7 | (-2.3, | 0.9) | 0.415 | -0.1 | (-1.2, | 1.1) | 0.916 |
| **Lower fat cheese (% TE)** | 0 | (-0.4, | 0.4) | 0.2 | (0.0, | 0.4) | 0.1 | (-0.1, | 0.3) | 0.2 | (-0.2, | 0.6) | 0.432 | 0.2 | (-0.2, | 0.7) | 0.275 | 0.1 | (-0.2, | 0.4) | 0.647 |
| **Higher fat yoghurt (% TE)** | -0.2 | (-0.9, | 0.5) | 0.2 | (-0.2, | 0.6) | -0.1 | (-0.5, | 0.3) | 0.5 | (-0.3, | 1.2) | 0.206 | 0.2 | (-0.5, | 1.0) | 0.589 | -0.3 | (-0.8, | 0.3) | 0.309 |
| **Lower fat yoghurt (% TE)** | 0 | (-0.3, | 0.4) | 0.2 | (0.0, | 0.4) | 0.5 | (0.2, | 0.7) | 0.2 | (-0.3, | 0.6) | 0.444 | 0.4 | (0.0, | 0.9) | 0.052 | 0.3 | (0.0, | 0.6) | 0.097 |
| **Higher fat spread (% TE)** | -0.6 | (-2.8, | 1.6) | -0.2 | (-1.4, | 1.1) | -0.7 | (-2.0, | 0.5) | 0.6 | (-1.7, | 2.9) | 0.625 | -0.5 | (-2.8, | 1.8) | 0.691 | -1 | (-2.7, | 0.6) | 0.217 |
| **Lower fat spread (% TE)** | -0.1 | (-0.6, | 0.5) | 0.4 | (0.1, | 0.7) | 0.4 | (0.0, | 0.7) | 0.4 | (-0.3, | 1.0) | 0.242 | 0.4 | (-0.2, | 1.1) | 0.182 | 0.1 | (-0.4, | 0.5) | 0.814 |
| **Lower fat ready meals (% TE)** | 0.3 | (-1.3, | 2.0) | -0.6 | (-1.5, | 0.4) | 0 | (-1.0, | 0.9) | -0.7 | (-2.0, | 0.7) | 0.582 | -0.6 | (-2.0, | 0.7) | 0.552 | 0 | (-0.9, | 1.0) | 0.956 |
| **Higher fat ready meals (% TE)** | -0.4 | (-2.2, | 1.4) | 0 | (-1.0, | 1.1) | -0.7 | (-1.7, | 0.4) | -0.5 | (-2.4, | 1.4) | 0.131 | -0.6 | (-2.4, | 1.3) | 0.653 | 0 | (-1.4, | 1.3) | 0.131 |
| **Salty snacks (% TE)** | 0.7 | (0.0, | 1.4) | -0.2 | (-0.6, | 0.2) | 0.1 | (-0.3, | 0.5) | -0.5 | (-1.2, | 0.2) | 0.316 | -0.2 | (-0.8, | 0.5) | 0.346 | 0.4 | (-0.1, | 0.8) | 0.938 |
| **Non-efficacy Outcomes** |  |  |  |  |  |  |  |  |  |  |  |  |  |  |  |  |  |  |  |  |  |
| **Energy density (kcal/g)** | -0.2 | (-0.3, | 0.0) | -0.1 | (-0.2, | 0.0) | 0 | (-0.1, | 0.1) | 0 | (-0.1, | 0.2) | 0.615 | 0.1 | (0.0, | 0.3) | 0.134 | 0.1 | (0.0, | 0.2) | 0.154 |
| **Total sugars (% TE)** | -2.3 | (-7.1, | 2.5) | 0.2 | (-2.6, | 3.0) | -0.1 | (-2.9, | 2.7) | 2.9 | (-1.3, | 7.2) | 0.175 | 2.7 | (-1.6, | 6.9) | 0.213 | -0.2 | (-3.3, | 2.8) | 0.879 |
| **Total sugars (kcal per £)** | -8.3 | (-27.0, | 10.4) | -4.1 | (-15.0, | 6.9) | -1.5 | (-12.4, | 9.5) | 4.6 | (-13.2, | 22.4) | 0.610 | 5.4 | (-12.4, | 23.3) | 0.547 | 0.9 | (-11.8, | 13.6) | 0.894 |
| **Fibre (g/1000 kcal)** | -0.7 | (-3.8, | 2.5) | 1.3 | (-0.5, | 3.2) | 1.4 | (-0.5, | 3.2) | 2 | (-1.7, | 5.7) | 0.291 | 2.6 | (-1.2, | 6.4) | 0.18 | 0.6 | (-2.1, | 3.3) | 0.660 |
| **Salt (g/100g purchased)** | -0.2 | (-0.6, | 0.2) | -0.1 | (-0.4, | 0.1) | -0.2 | (-0.5, | 0.0) | -0.2 | (-0.5, | 0.1) | 0.125 | -0.1 | (-0.4, | 0.1) | 0.378 | 0.1 | (-0.1, | 0.3) | 0.352 |
| **Absolute basket cost (£/week)** | -1.4 | (-13.3, | 10.5) | 1.2 | (-5.7, | 8.2) | 2.6 | (-4.4, | 9.5) | 5.2 | (-5.1, | 15.4) | 0.317 | 5.5 | (-4.8, | 15.8) | 0.29 | 0.3 | (-7.0, | 7.6) | 0.929 |
| **Relative basket cost (£/Kg)** | 0 | (-0.5, | 0.5) | -0.3 | (-0.5, | 0.0) | 0.1 | (-0.2, | 0.4) | -0.2 | (-0.7, | 0.2) | 0.311 | 0.1 | (-0.4, | 0.5) | 0.812 | 0.3 | (0.0, | 0.6) | 0.081 |
| *** Estimates from linear regression models adjusting for GP practice and baseline, all p values >0.05; ** TE: Total energy from purchases** | | | | | | | | | | | | | | | | | | | | | |
|  |  |  |  |  |  |  |  |  |  |  |  |  |  |  |  |  |  |  |  |  |  |

## Table E. Blood lipids, blood pressure and weight (means (standard deviations)).

|  | **Control (n=15)**  **Mean (SD)** | | | | **Brief Support (n=44)**  **Mean (SD)** | | | | **Brief Support plus Shopping Advice (n=44)**  **Mean (SD)** | | | |
| --- | --- | --- | --- | --- | --- | --- | --- | --- | --- | --- | --- | --- |
|  | **Baseline** | | **Follow up** | | **Baseline** | | **Follow up** | | **Baseline** | | **Follow up** | |
| LDL-cholesterol | 3.95 | 0.46 | 3.80 | 0.56 | 4.10 | 0.59 | 3.71 | 0.53 | 3.99 | 0.76 | 3.86 | 0.67 |
| HDL-cholesterol | 1.51 | 0.50 | 1.60 | 0.56 | 1.40 | 0.39 | 1.39 | 0.35 | 1.37 | 0.34 | 1.35 | 0.36 |
| Total cholesterol | 6.10 | 0.47 | 5.98 | 0.40 | 6.17 | 0.72 | 5.71 | 0.62 | 6.08 | 0.92 | 5.94 | 0.77 |
| Triglycerides | 1.41 | 0.71 | 1.26 | 0.77 | 1.48 | 0.66 | 1.34 | 0.52 | 1.67 | 1.20 | 1.67 | 1.17 |
| Non-HDL cholesterol | 4.59 | 0.62 | 4.38 | 0.77 | 4.77 | 0.72 | 4.32 | 0.63 | 4.71 | 0.91 | 4.59 | 0.78 |
| Total cholesterol/HDL ratio | 4.44 | 1.47 | 4.29 | 1.81 | 4.67 | 1.19 | 4.33 | 1.05 | 4.71 | 1.38 | 4.74 | 1.50 |
| Systolic blood pressure (mmHg) | 130.0 | 11.8 | 130.5 | 11.6 | 130.7 | 20.0 | 131.9 | 19.7 | 132.6 | 15.2 | 133.8 | 17.8 |
| Diastolic blood pressure (mmHg) | 76.7 | 7.7 | 77.9 | 7.9 | 76.9 | 11.0 | 76.6 | 10.3 | 82.1 | 9.0 | 81.6 | 10.4 |
| Weight (kg) | 73.2 | 16.1 | 72.9 | 16.0 | 74.9 | 14.9 | 73.8 | 14.7 | 79.4 | 18.4 | 78.6 | 19.4 |
| *LDL= low density lipoprotein cholesterol; HDL= high density lipoprotein cholesterol; | | | | | | | | | | | | |

## Table F. Changes in SFA from dietary recalls (%Energy Intake), loyalty card data (%Total Energy) and LDL cholesterol (mmol/L) by group and education.

|  | Change from baseline within group | | | | | | | | | Between-group differences adjusted for baseline and practice | | | | | | | | | | | |
| --- | --- | --- | --- | --- | --- | --- | --- | --- | --- | --- | --- | --- | --- | --- | --- | --- | --- | --- | --- | --- | --- |
|  | **Control** | | | **Brief Support (BS)** | | | **Brief Support plus**  **Shopping Feedback (SF)** | | | **BS vs control** | | | | **SF vs control** | | | | **SF vs BS** | | | |
| Primary Outcome | Mean | 95% CI | | Mean | 95% CI | | Mean | 95% CI | | Mean | 95% CI | | P value | Mean | 95% CI | | P value | Mean | 95% CI | | P value |
| Saturated fat (% EI)* |  |  |  |  |  |  |  |  |  |  |  |  |  |  |  |  |  |  |  |  |  |
| <=Secondary education** | -0.3 | (-3.0, | 2.4) | -1.1 | (-2.4, | 0.2) | -0.9 | (-2.2, | 0.5) | -1.5 | (-4.1, | 1.1) | 0.254 | -0.6 | (-3.2, | 2.0) | 0.630 | 0.9 | (-0.8, | 2.5) | 0.302 |
| > Secondary education | -0.1 | (-2.6, | 2.5) | 0.1 | (-1.7, | 1.9) | -1.0 | (-2.7, | 0.8) | 1.1 | (-1.6, | 3.7) | 0.434 | 0.1 | (-2.6, | 2.8) | 0.953 | -1.0 | (-3.1, | 1.1) | 0.362 |
| Secondary Outcomes | Mean | 95% CI | | Mean | 95% CI | | Mean | 95% CI | | Mean | 95% CI | |  | Mean | 95% CI | |  |  | 95% CI | |  |
| Saturated fat (% of TE) |  |  |  |  |  |  |  |  |  |  |  |  |  |  |  |  |  |  |  |  |  |
| <=Secondary education | 0.5 | (-2.0, | 3.1) | -1.6 | (-2.8, | -0.3) | -0.9 | (-2.3, | 0.4) | -1.9 | (-4.3, | 0.6) | 0.129 | -1.2 | (-3.7, | 1.2) | 0.319 | 0.6 | (-0.9, | 2.2) | 0.420 |
| > Secondary education | -1.5 | (-3.8, | 0.9) | -0.9 | (-2.6, | 0.7) | -2.2 | (-3.8, | -0.7) | 0.5 | (-2.0, | 2.9) | 0.713 | -0.4 | (-2.9, | 2.0) | 0.737 | -0.9 | (-2.9, | 1.1) | 0.380 |
| LDL-cholesterol (mmol/L) |  |  |  |  |  |  |  |  |  |  |  |  |  |  |  |  |  |  |  |  |  |
| <=Secondary education | -0.06 | (-0.57, | 0.46) | -0.47 | (-0.73, | -0.22) | -0.24 | (-0.50, | 0.02) | -0.19 | (-0.66, | 0.29) | 0.440 | 0.03 | (-0.45, | 0.50) | 0.914 | 0.21 | (-0.09, | 0.51) | 0.167 |
| > Secondary education | -0.28 | (-0.75, | 0.20) | -0.26 | (-0.60, | 0.08) | 0.03 | (-0.30, | 0.36) | -0.08 | (-0.57, | 0.40) | 0.734 | 0.09 | (-0.39, | 0.58) | 0.701 | 0.18 | (-0.21, | 0.57) | 0.366 |
| * Estimates from linear regression models adjusting for GP practice and baseline; all *p* values for the interaction group x education were >0.05;  ** <=Secondary education: control n=7; BS n=28; SF n=26; and > Secondary education: control n=8; BS n=16; SF n=17  *** EI = energy intake; TE = total energy; LDL= low density lipoprotein cholesterol; | | | | | | | | | | | | | | | | | | | | |  |

## Table G. Sensitivity analyses

Sensitivity analyses on the primary outcome measure: a) using baseline observation carried forward for participants who had missing data for the outcome; b) excluding participants who had one dietary recall or implausible dietary intakes; c) excluding participants who received their intervention session >45 days from baseline; d) excluding participants who changed their relevant medications during the study; e) adjusting for BMI.

|  | Change from baseline within group | | | | | | | | | Between-group differences adjusted for baseline and practice | | | | | | | | | | | |
| --- | --- | --- | --- | --- | --- | --- | --- | --- | --- | --- | --- | --- | --- | --- | --- | --- | --- | --- | --- | --- | --- |
|  | **Control** | | | **Brief Support (BS)** | | | **Brief Support plus**  **Shopping Feedback (SF)** | | | **BS vs control** | | | | **SF vs control** | | | | **SF vs BS** | | | |
| Primary Outcome  Saturated fat (% EI)* | Mean | 95% CI | | Mean | 95% CI | | Mean | 95% CI | | Mean | 95% CI | | P value | Mean | 95% CI | | P value | Mean | 95% CI | | P value |
| Missing outcome data n=113 | -0.1 | -1.7 | 1.6 | -0.7 | -1.7 | 0.3 | -0.8 | -1.8 | 0.2 | -0.4 | -2.1 | 1.4 | 0.686 | -0.2 | -1.9 | 1.6 | 0.846 | 0.2 | -1.1 | 1.4 | 0.775 |
| Missing one dietary recall n=101 | 0.5 | -1.3 | 2.3 | -0.7 | -1.8 | 0.4 | -0.9 | -2.0 | 0.2 | -0.8 | -2.6 | 1.0 | 0.398 | -0.6 | -2.4 | 1.2 | 0.515 | 0.2 | -1.1 | 1.5 | 0.794 |
| Dropping implausible dietary recalls n=104 | 0.2 | -1.6 | 2.0 | -0.7 | -1.8 | 0.3 | -0.9 | -2.0 | 0.2 | -0.5 | -2.4 | 1.3 | 0.567 | -0.3 | -2.2 | 1.6 | 0.738 | 0.2 | -1.1 | 1.6 | 0.753 |
| Dropping late intervention n=83 | -0.1 | -1.9 | 1.8 | -0.7 | -2.0 | 0.6 | -0.9 | -2.3 | 0.4 | -0.4 | -2.3 | 1.6 | 0.705 | 0.1 | -1.9 | 2.1 | 0.948 | 0.4 | -1.1 | 2.0 | 0.584 |
| Change medications n=104 | -0.1 | -1.8 | 1.7 | -0.6 | -1.6 | 0.4 | -1.0 | -2.1 | 0.1 | -0.3 | -2.1 | 1.5 | 0.767 | -0.2 | -2.0 | 1.7 | 0.860 | 0.1 | -1.2 | 1.4 | 0.874 |
| BMI adjusted model n=106 | -0.5 | -2.1 | 1.0 | -0.8 | -1.7 | 0.1 | -0.6 | -1.5 | 0.3 | -0.3 | -2.1 | 1.5 | 0.718 | -0.1 | -1.9 | 1.7 | 0.917 | 0.2 | -1.1 | 1.5 | 0.728 |
| * EI = energy intake  **n=7 participants had missing data for the primary outcome, BMI adjusted analysis was proposed post-hoc given the small imbalance observed in BMI at baseline | | | | | | | | | | | | | | | | | | | | | |

## Table H. Changes in motivation, knowledge and attitudes, and other process evaluation measures

| . | **All (n=113)** | | **Control (n=17)** | | **BS (n=48)** | | **SF (n=48)** | |
| --- | --- | --- | --- | --- | --- | --- | --- | --- |
| **Aceptability of the intervention components - Average score 1-5** |  |  |  |  |  |  |  |  |
| Brief advice session with the HCP to motivate change (mean, SD) | N/A | N/A | N/A | N/A | 3.9 | 1.2 | 3.7 | 1.2 |
| Leaflets provided during the advice session (mean, SD) | N/A | N/A | N/A | N/A | 4.1 | 0.9 | 3.7 | 1.1 |
| Shopping report provided monthly throughout the study period (mean, SD) | N/A | N/A | N/A | N/A | N/A | N/A | 3.5 | 1.2 |
| **Knowledge and Behaviour Change** |  |  |  |  |  |  |  |  |
| Change in % participants who think SFA affects CVD | +8% | N/A | +6% | N/A | +13% | N/A | +2% | N/A |
| Change in % participants who have tried to reduce SFA intake in the last 3 months | +49% | N/A | +50% | N/A | +68% | N/A | +30% | N/A |
| Change in % participants who are very/somewhat confident of major sources of SFA in their diet | +25% | N/A | +13% | N/A | +30% | N/A | +23% | N/A |
| **Grocery shopping behaviours** |  |  |  |  |  |  |  |  |
| Number of trips/week before baseline (mean, SD) | 1.4 | 1.1 | 1.6 | 0.9 | 1.4 | 1.3 | 1.2 | 0.7 |
| Number of trips/week after baseline (mean, SD) | 1.4 | 1.0 | 1.7 | 1.0 | 1.5 | 1.0 | 1.4 | 0.9 |
| £ spent/week before baseline (mean, SD) | 36.4 | 34.6 | 34.6 | 25.6 | 37.4 | 43.7 | 36.0 | 26.0 |
| £ spent/week after baseline (mean, SD) | 37.4 | 24.8 | 31.7 | 20.5 | 39.5 | 25.7 | 37.2 | 25.3 |
| £ spent/trip before baseline (mean, SD) | 29.2 | 18.1 | 22.2 | 13.3 | 30.2 | 19.1 | 30.5 | 18.3 |
| £ spent/trip after baseline (mean, SD) | 28.4 | 16.6 | 21.3 | 12.9 | 30.1 | 18.1 | 29.1 | 15.8 |
| **Swap behaviours** (% of swaps offered from different food groups): |  |  |  |  |  |  |  |  |
| Cakes, biscuits, desserts | N/A | N/A | N/A | N/A | N/A | N/A | 21% | N/A |
| Meat | N/A | N/A | N/A | N/A | N/A | N/A | 17% | N/A |
| Cheese | N/A | N/A | N/A | N/A | N/A | N/A | 13% | N/A |
| Milk, cream | N/A | N/A | N/A | N/A | N/A | N/A | 13% | N/A |
| Yoghurt | N/A | N/A | N/A | N/A | N/A | N/A | 3% | N/A |
| Spread | N/A | N/A | N/A | N/A | N/A | N/A | 12% | N/A |
| Ready meals (e.g. pizza) | N/A | N/A | N/A | N/A | N/A | N/A | 7% | N/A |
| Salty snacks | N/A | N/A | N/A | N/A | N/A | N/A | 2% | N/A |
| Number of items from those offered purchased over the intervention period: |  |  |  |  |  |  |  |  |
| % of TOTAL swaps accepted out of those offered over the intervention period | N/A | N/A | N/A | N/A | N/A | N/A | 25% | N/A |
| % of swaps accepted from cakes, biscuits, desserts | N/A | N/A | N/A | N/A | N/A | N/A | 15% | N/A |
| % of swaps accepted from meat | N/A | N/A | N/A | N/A | N/A | N/A | 13% | N/A |
| % of swaps accepted from cheese, yoghurt, milk, cream | N/A | N/A | N/A | N/A | N/A | N/A | 43% | N/A |
| % of swaps accepted from spread | N/A | N/A | N/A | N/A | N/A | N/A | 15% | N/A |

*healthcare professional - HCP; standard deviation - SD; cardiovascular disease - CVD; saturated fat - SFA
