# Supplementary material for: Evaluation of an intervention to provide brief support and personalized feedback on food shopping to reduce saturated fat intake (PC-SHOP): A randomized controlled trial
Source: PLoS Med. 2020 Nov 5;17(11):e1003385. doi: 10.1371/journal.pmed.1003385 (PMC7643942; doi:10.1371/journal.pmed.1003385)
Supplement: Attachment — Submitted filename: PMEDICINE responses to review 20 02 2020 final.docx [file pmed.1003385.s006.docx]

PMEDICINE-D-19-03921

-----------------------------------------------------------

Requests from the editors:

General comment: Please add a space before reference brackets

**Answer: We have corrected this**

Data Availability Statement: "PLOS Medicine requires that the de-identified data underlying the specific results in a published article be made available, without restrictions on access, in a public repository or as Supporting Information at the time of article publication, provided it is legal and ethical to do so. Please see the policy at <http://journals.plos.org/plosmedicine/s/data-availability> and FAQs at

<http://journals.plos.org/plosmedicine/s/data-availability#loc-faqs-for-data-policy>

If the data are not freely available, as you have stated, please describe briefly the ethical, legal, or contractual restriction that prevents you from sharing it. Please also include an appropriate contact (web or email address) for inquiries (please note that this cannot be a study author)

**Answer: We have clarified our Data Availability Statement to state that the datasets used and/or analysed during the current study contain potentially sensitive participant information, and will be available from the corresponding author on reasonable request. Data on food shopping which was used in our secondary outcome analysis was provided by the collaborating retailer under contractual agreement which prevents making this data available outside the research team. We have clarified this as well in the Data Availability Statement.**

**Data Availability Statement:**

**“This study includes potentially sensitive participant information data collected by the research team from March 2018 to January 2019, which includes information on the clinical measurements, health conditions, medications, and dietary intake measurements (including the primary outcome measure). Anonymised version of this data is available from the corresponding author on reasonable request. This study also includes grocery shopping data from the collaborating supermarket which was received weekly from March 2018 to January 2019. Grocery shopping data was supplemented with a dataset containing the nutritional composition of foods provided by a commercial organisation (Brand View). Because of contractual restrictions to the use of the grocery shopping data it is not possible to make this data openly available outside this project”**

Please revise your title according to PLOS Medicine's style, placing the study design in the subtitle (ie, after a colon). We suggest “Evaluation of an intervention to provide brief support and personalised feedback on food shopping and saturated fat intake (PC-SHOP): a randomised controlled trial” or similar.

**Answer: We have modified our title as suggested**

Please report your abstract according to CONSORT for abstracts, following the PLOS Medicine abstract structure (Background, Methods and Findings, Conclusions) <http://www.consort-statement.org/extensions?ContentWidgetId=562>

**Answer: We have revised our abstract and added the required information following the CONSORT guidance and the journal’s suggested structure**

Abstract Background: Please expand upon the context of why the study is important.

**Answer: We have added a sentence to state the relevance of the study**

**Revised text:**

**Guidelines recommend reducing saturated fat (SFA) intake to decrease cardiovascular disease (CVD) risk but there is limited evidence on scalable and effective approaches to change dietary intake given the large proportion of the population exceeding SFA recommendations.**

Abstract Methods and Findings:

Please include brief demographic details of the participants (e.g. age, sex, etc) and further details of the study setting (e.g. locations), number of participants, dates between which the study took place and length of follow up.

Please state that analysis was intention to treat.

Please quantify the main results (with 95% CIs and p values).

In the last sentence of the Abstract Methods and Findings section, please describe the main limitation(s) of the study's methodology.

**Answer: We have added the requested details to the methods section of the abstract**

**Revised text:**

**Methods and findings**

The Primary Care Shopping Intervention for Cardiovascular Disease Prevention (PCSHOP) study is a parallel randomised controlled trial with a 3 month follow-up conducted between March 2018 to January 2019. Adults ≥18 years with LDL-cholesterol>3mmol/L (n=113) were recruited from GP practices in Oxfordshire and randomly allocated to: ‘Brief Support’ (BS, n=48), ‘Brief Support+Shopping Feedback’ (SF, n=48) or ‘Control’ (n=17). BS consisted of a 10-minute consultation with a HCP to motivate participants to reduce their SFA intake. Shopping feedback comprised a personalised report on the SFA content of grocery purchases and suggestions for lower-SFA swaps. The primary outcome was the between-group difference in change in SFA intake (% total energy intake) at three months adjusted for baseline SFA and GP practice using intention-to-treat analysis. Secondary outcomes included %SFA in purchases, LDL cholesterol and feasibility outcomes. The trial was powered to detect an absolute reduction in SFA of 3%(SD3). Neither participants nor the study team were blinded to group allocation.

A total of 106 (94%) participants completed the study. There were small decreases in SFA intake at 3 months: control= -0.1%[95%CI -1.8,1.7], BS= -0.7%[95%CI -1.8,0.3], SF= -0.9%[95%CI -2.0,0.2]; but no evidence of a significant effect of either intervention compared to control (adjusted difference: BS vs control= -0.33% [95%CI -2.11, 1.44]; SF vs control= -0.11% [95%CI -1.92, 1.69]). There were similar trends in %SFA based on supermarket purchases: control= -0.5% [95%CI -2.3,1.2], BS= -1.3% [95%CI -2.3,-0.3], SF= -1.5% [95%CI -2.5,-0.5] from baseline to follow-up, without differences between intervention and control group. There were small reductions in LDL from baseline to follow up (control = -0.14mmol/L [95%CI -0.48, 0.19), BS: -0.39mmol/L [95%CI -0.59, -0.19], SF:-0.14mmol/L [95%CI -0.34, 0.07]), but these were not significantly different from control. Limitations of this study include the small sample of participants recruited which limits the power to detect smaller differences and the low response rate (3%) which may limit the generalisability of these findings.

Please begin your Abstract Conclusions with “"In this study, we observed ..." or similar. Please address the study implications, emphasizing what is new without overstating your conclusions.

**Answer: We have modified our conclusions as suggested**

**Revised text:**

**Conclusions**

In this study, we have shown it is feasible to deliver brief advice in primary care to encourage reductions in SFA intake and to provide personalised advice to encourage healthier choices using supermarket loyalty card data. There was no evidence of large reductions in SFA but we are unable to exclude more modest benefits. The feasibility, acceptability and scalability of these interventions suggest they have potential to encourage small changes in diet which could be beneficial at the population level.

At this stage, we ask that you include a short, non-technical Author Summary of your research to make findings accessible to a wide audience that includes both scientists and non-scientists. The Author Summary should immediately follow the Abstract in your revised manuscript. This text is subject to editorial change and should use non-identical language distinct from the scientific abstract. Please see our author guidelines for more information: <https://journals.plos.org/plosmedicine/s/revising-your-manuscript#loc-author-summary>

**Answer: We have included a non-technical summary of our results in our revised manuscript**

**Author Summary**

**Why Was This Study Done?**

Cardiovascular disease (CVD) is the leading cause of death in the UK and is strongly influenced by diet composition. Reducing the intake of saturated fat (e.g. fats from animal sources such as butter or meat), mostly by swapping some key foods in the diet for others that are lower in saturated fat (SFA), can help reduce the ‘bad’ low-density lipoprotein (LDL)-cholesterol in the blood, and reduce the risk of CVD.

Previous studies have achieved success either by providing particular foods to people or by giving them intensive support and advice from nutrition specialists. Currently, there are no practical interventions shown to help large numbers of people improve their diet to reduce the amount of saturated fat they eat.

**What Did the Researchers Do and Find?**

In this study, we developed a system to provide regular information on the saturated fat content of food purchases and suggest healthier swaps using loyalty card data from the UK largest grocery store. Participants received brief oral and written advice from a nurse or healthcare professional (HCP) at their general practitioner (GP) practice alone or in combination with personalised feedback on their food shopping. Our primary aim was to test if this approach was effective to decrease saturated fat intake compared to usual care which does not involve any specific advice. We also compared changes in LDL cholesterol and the quality of the grocery shopping. We recruited adults who had a blood test showing they had raised LDL cholesterol from GP practices in Oxfordshire (UK) and we followed them for approximately 3 months.

We found small decreases in SFA intake as well as the SFA content of food purchases and reductions in LDL cholesterol but these changes were not significantly different from those observed in the control group. Participants reported positive feedback regarding the brief advice and the personalised feedback on their food shopping which they received monthly throughout the study.

**What Do These Findings Mean?**

Previous studies have shown that self-monitoring and feedback are effective strategies to help people change their behaviour. In this study we were able to use data from supermarket loyalty cards to provide regular feedback and healthier swaps to help people improve the quality of their grocery shopping. Participants valued and used this information together with the brief advice received from primary care practitioners to reduce their saturated fat intake.

The trial was designed to detect a clinically significant difference in SFA intake between groups of 3% and the intervention did not achieve such large effects. However, modelling studies suggest that just replacing 1% of saturated fat with polyunsaturated fat can potentially lead to an 8% reduction in CVD events. With future development and testing this may be an intervention which could be offered by supermarkets to achieve small improvements in diet with population-level health benefits.

Introduction

Please further address past research and explain the need for and potential importance of your study. If there has been a systematic review of the evidence related to your study (or you have conducted one), please refer to and reference that review and indicate whether it supports the need for your study.

Line 80 - please add ‘to our knowledge...’ or similar, to avoid assertions of primacy.

**Answer: We have tried to emphasize the importance of this work given the high intakes of SFA in the UK and highlighted the lack of brief, scalable and effective interventions that can help people reduce SFA intake. We included evidence from previous trials or systematic reviews which show that effectiveness in reducing SFA has been only achieved with high intensity behavioural support and provision of lower SFA foods. We also changed line 80.**

**Revised text:**

SFA intake in the UK population (13.5% total energy) and in other higher income countries remains higher than the dietary recommendation of <10% of the total energy intake, and it is estimated that >70% of the UK population exceeds this level. Systematic reviews and recent trials have shown that reductions in dietary SFA intake can lower LDL cholesterol but this has only been achieved with specialist staff and high intensity behavioural support as well as with the provision of appropriate food substitutes in place of higher SFA products. There is evidence that brief behavioural interventions delivered in routine primary care can help people engage in weight loss programmes with the potential to achieve population-level impact. However, there is limited evidence of brief interventions that are effective for improving the nutritional quality of diet and sufficiently scalable for routine delivery in primary care settings by generalist staff, or at the population level

Methods

Please complete the CONSORT checklist and ensure that all components of CONSORT are present in the manuscript. When completing the checklist, please use section and paragraph numbers, rather than page numbers

**Answer: We have completed and attached a new CONSORT checklist with line numbers**

With regard to your prospective protocol and analysis plan, please make sure that the Methods section transparently describes when analyses were planned, and whether or not any data-driven changes to analyses took place (indicating what those are)

**Answer: We have clarified this in our Methods section (lines 162-166)**

**Revised text:**

The trial protocol includes further information regarding the trial design and interventions [22]. A protocol and statistical analysis plan were prospectively registered at ISRCTN14279335 in September 2017 and March 2019 respectively, with prospective changes introduced to reflect delays in the planned recruitment and follow up dates as well as the addition of a qualitative sub-study and a few exploratory outcomes.

Line 96 - please clarify whether informed consent was written or oral

**Answer: We have clarified this (line 158)**

Line 102 - please specify how many primary care practices were involved

**Answer: We have clarified this (line 168)**

Line 209-212 - please expand on the methods used to assess these outcomes

**Answer: We have clarified this**

**Revised text:**

For the process evaluation, we assessed the feasibility of recruitment and follow up by measuring the number of participants who accepted the invitation and consented to take part as well as the number of participants that completed follow up. We measured acceptability of the intervention through questionnaires completed at baseline and follow up (rated on a scale 1-5 from least to most helpful). The fidelity of the intervention delivery by the HCP was measured through the analysis of audio-taped sessions.

Line 214 - please define SD (to avoid confusion with standard deviation)

**Answer: We were referring to standard deviation but we have spelled this out (line 304)**

Line 221 - please clarify what is meant by ‘2% (3% SD)’

**Answer: We have clarified this (line 312)**

Line 230 - Please provide a copy of the questionnaires used in your supplementary information.

**Answer: We have included samples of the questionnaires in supplementary information.**

Results

Please provide p values in addition to 95% CIs throughout your results section.

**Answer: We have included the p values in addition to the CI where we presented the differences between each intervention arm vs control.**

Please include Figure S1 in the main text of your manuscript.

**Answer: We have included Figure S1 in the main manuscript, now as Figure 1**

Please define the length of follow up (eg, in mean, SD, and range).

**Answer: We have clarified this in the text.**

**Revised text:**

Follow up was completed on the 16th of January 2019 and 106 (94%) participants completed the study after an average of 115 days (SD 26, min 77, max 208) (Figure 1).

Line 261 - Please indicate which factors are adjusted for

**Answer: We have clarified that differences were adjusted for GP practice and baseline value (line 358-359).**

Line 275 - please revise ‘somewhat greater reductions’ to clarify that reductions were non-significant

**Answer: We have clarified this**

Line 306 - please revise ‘slightly larger reductions’ to clarify that reductions were non-significant

**Answer: We have clarified this**

Line 356 - please replace % with ‘percentage’, ‘proportion’ or similar

**Answer: We have corrected this**

Discussion

Line 369 - please revise ‘somewhat greater reductions’ to clarify that reductions were non-significant

**Answer: We have clarified this to indicate these changes were not significant**

Line 450 - please clarify which groups are being compared when discussing these results

**Answer: We have clarified this (line 468)**

Line 482 - please avoid using the word ‘novel’, in order to avoid assertions of primacy

**Answer: We have clarified this**

Figure 1 - please define SFA and SEM in the figure legend

**Answer: We have clarified this in the main outcome figure**

Figure 2 - please define EI and TE in the figure legend

**Answer: We have clarified this in the figure**

Figure 3 - please define EI, TE, SFA, BS and SF, LDL in the figure legend

**Answer: We have clarified this in the figure**

Supplementary Tables - please define all abbreviations used in the table legend

Table S6 - When a p value is given, please specify the statistical test used to determine it.

**Answer: We have defined all abbreviations in tables and statistical tests where relevant.**

References

Please ensure that all references are appropriately formatted and capitalised e.g. refs 7, 25, 27, 28, 29, 31, 33

**Answer: We have reviewed all references to make sure the formatting is appropriate**

-----------------------------------------------------------

Comments from the reviewers:

Reviewer #1: Thank you for the opportunity to review your manuscript. This study presents the findings of a 3-arm RCT that tests whether there are changes in saturated fat intake (% total energy) when patients with high LDL cholesterol are exposed to usual care, brief support, and brief support with enhanced feedback on shopping purchases. My role is as a statistical reviewer and I have focused on the design and analysis. The protocol and statistical analysis plan are included, the supplementary material attached to this publication is well-organised and I found it helpful when conducting my review. I have previously collaborated with colleagues working in general practice, primary prevention, and food databases and I appreciate the thoughtfulness and potential of this work.

**Answer: We appreciate the reviewer’s comments and have tried to address and their concerns regarding the statistical methods reported in this manuscript.**

P6, Line 114. A 1:3:3 allocation ratio is used, but there doesn't appear to be a justification for this in the protocol or manuscript. Was there a specific reason that the usual practice control group had a smaller allocation?

**Answer: We apologise for the lack of clarity regarding the allocation ratio used in this study. Our primary outcome compared the change in SFA between each intervention and control (lines 307-309). The difference that we wanted to detect was 3% (SD 3), therefore we needed 16 participants per group. However, we also stated that a secondary comparison (lines 309-311) was to compare the 2 active intervention arms to each other to detect a further difference of 2% (SD 3) between them, for which we needed 48 participants in each of the active arms to account for 10% drop outs, 90% power and multiple comparisons. This was the most efficient way of allowing the necessary power for each of the proposed comparisons.**

**We have rewritten this paragraph to improve its clarity:**

We calculated a total sample of 112 (16 control, 48 BS and 48 SF group) using intention-to-treat analysis with 90% power, 10% attrition and statistical adjustment for multiple comparisons (using two-sided α=0.01 in the primary outcome analysis). Using a 1:3:3 allocation ratio we calculated a sample of 48 participants in each active intervention arms to have 90% power to detect a further difference in %SFA intake between BS and SF interventions of 2% (3% standard deviation). All the other tests were performed at a 5% two-sided significance level, Stata 14 was used for the analyses.

P6, Line 116. The protocol (p21) states that randomly varying block sizes were used, but then specifies that all the blocks were of size 7. Can you confirm whether they were randomly sized, or used a fixed block size with random order?

**Answer: Apologies for the lack of clarity, we used fixed block sizes of 7 with random order to ensure even allocation. We have corrected this mistake in our protocol and manuscript (line 190).**

P10, L213. 'Statistically significant' is used throughout the reporting of results, but there are not details as to what was statistically significant in the methods (e.g. alpha < 0.05, 95% CI overlaps null effect?). If 'statistically significant' is to be used, then this should be formally defined, if based on a p-value then this should be presented.

**Answer: We apologise for missing this important part of information which was originally stated in our protocol. We have included this information in our revised manuscript (See the rewritten paragraph above).**

P10, L214 Where was the standard deviation for % of SFA for the sample size calculations taken from?

**Answer: We apologise for omitting this information which was originally included in our protocol. We used two intervention studies to guide our effect size and SD, but considering the differences (intervention intensity, length of follow up) between those studies and ours, we powered our study for an absolute difference between intervention and control groups of 3% in SFA with SD 3%. We have included references and have rewritten this paragraph in our manuscript to clarify this (lines 304-305).**

P10, L219. In the power calculation it says that a multiplicity adjustment is included, but there are no details of what the selected multiplicity adjustment was that was incorporated into the sample size calculation, and whether a multiplicity adjustment was applied to the final analysis (and what it was).

**Answer: We adjusted for multiple comparisons at the time of calculating the sample size by reducing the p value to 0.01. We have clarified this in our manuscript (lines 309, see paragraph above).**

P13, Table 1. Just confirming the results for BMI by category vs. average BMI. There is very little difference between groups in average BMI, but when reported as % per categories there appears to be some variation between groups.

**Answer: The results for the BMI categories are correct, there was a small imbalance between the trial arms in the average BMI and BMI categories. This is something that we didn’t anticipate and so we did not plan to adjust our analyses for BMI. However, we have explored a post-hoc adjustment for BMI in the model and have seen negligible changes to the estimates for the analysis of the main outcome which don’t change the interpretation of the reported results (see Supplemental Table S7).**

P21, L226. The primary analysis not only includes an adjustment for baseline value, but also for practice. Just to clarify, was the practice included as a fixed effect or as a random effect in the main regression model?

**Answer: We used a general linear (non-** **hierarchical) model so adjustment for GP practice was fixed.**

P22, L318. The most powerful test of differences between subgroups showed no important differences between groups, but the last sentence gives the impression that there was a useful difference found - I think the wording should be adjusted to reflect this. Also, if a p-value is used as evidence for or against an interaction, it should be presented.

**Answer: We apologise for this misinterpretation of the results and have corrected our language to avoid implying that there was an interaction effect.**

P24, L326. The results of the sensitivity analyses should be included in the supplementary materials.

**Answer: We have included a new Supplemental Table S7 showing the results of the sensitivity analysis which were only performed for the primary outcome analysis.**

Consort checklist: Have the page numbers reported in the checklist shifted when it was formatted for submission? I couldn't find some of the items (e.g. eligibility and trial design should be on p4 and p3) in the manuscript in the pages they are reported.

**Answer: We have submitted another CONSORT checklist and checked that all numbers were correct.**

-----------------------------------------------------------

Reviewer #2:

Thank you for the opportunity to read this interesting paper reporting a well-conducted 3-arm hybrid trial examining changes in SFA intake after a behavioural intervention. The retention rate is excellent and the finding that no significant between-group differences in changes emerged over this timescale in this particular sample size is an important one to inform future work. I have some suggestions to further clarify aspects of the manuscript, which the authors may like to consider.

**Answer: We thank the reviewer for their thoughtful comments on our manuscript, we have tried to address all their concerns to improve the clarity and presentation of the results.**

1. Typo in short title - 'amd' should be 'and'

**Answer: We have corrected this**

Abstract

2. Would the authors consider mentioning that that RCT was a hybrid trial in the Abstract? Also, in the abstract it may help the reader to mention the retention rate / note the final N and to note that the primary outcome came from self-reported 24hr recalls of intake.

**Answer: This trial was conceived as an effectiveness trial but included feasibility outcomes given the lack of evidence on the feasibility of using loyalty card data to provide personalised feedback. We have indicated the hybrid effectiveness-feasibility design in the abstract and the manuscript where appropriate. We have also included the retention rate in the abstract as requested.**

3. The values for adjusted between group differences do not appear to be reported in the Abstract, although this is stated as the primary outcome in the Abstract itself. Unless I have misunderstood this, these adjusted values should be reported as well as - or instead of - the absolute, unadjusted % SFA intake changes currently reported.

**Answer: We have added the adjusted between group differences with their 95%CI around it.**

Method

4. Were outcome assessors of the variables that could not be reported via online portal blinded to allocation - for example, body weight? I assume that this is what is referred to when talking about blinding at follow up, but this could benefit from clarification.

**Answer: The reviewer is right and some of the secondary outcomes (clinical measurements including body weight, blood pressure, point of care blood lipid measures) were taken by unblinded research staff. We have clarified this in our methods section (Line 200).**

5. I found myself searching for some of the information contained in supplementary material in order to understand the basics of the RCT. A key example of this was participant exclusion/inclusion based on sufficient use (or not) of the specific supermarket chain. Is there scope to include more details about inclusion/exclusion in the main body of the Method?

**Answer: We have included the inclusion/exclusion criteria in the main body of the manuscript.**

6. Could data for the numbers of participants who did not respond to the invitation / who were screened ineligible prior to consent be added to the CONSORT diagram at the first stage - this is not essential but provides additional interesting information that is presently presented in the text?

**Answer: We have added this information to the CONSORT diagram.**

7. Please consider whether it would be appropriate to provide brief extra behavioural details of the intervention delivered (e.g. 'box 1' as shown in Lombard et al, 2016; <https://journals.plos.org/plosmedicine/article?id=10.1371/journal.pmed.1001941>). I realise that details of the intervention are provided in the protocol paper, but a very brief summary would be immensely helpful to behaviourally-oriented readers.

**Answer: We thank the reviewer for this helpful suggestion. We have included a box with brief details of the behavioural intervention delivered in this trial.**

8. Please could the rationale for presenting both 24 hr recall and supermarket data be more clearly stated?

**Answer: We have stated our rationale more clearly in our methods section (Line 270-272).**

Results

9. Please consider whether figure and table notes need to define acronyms like SFA and EI.

**Answer: We thank the reviewer and have included all definitions in the footnotes.**

10. As it took on average 37 days from baseline to intervention delivery, would the authors also consider reporting the mean number of days between receiving the intervention and completing follow-up assessment? Did the 3 month period start from the day of the baseline assessment or the day of the intervention appointment? How did this month-long average delay affect the number of shopping feedback components delivered to participants in this group - did all participants receive all 4 sets of feedback (as is suggested by an average of 3.6 in the SF group)?

**Answer: We appreciate this comment and have tried to clarify this in our results section as the editor also noted this (line 342). The 3-month period was originally planned from the date of randomisation, however the average time in the study from baseline to follow up was 115 days (SD 26). This is because where there was a delay in receiving their first intervention session with the health care practitioner (due to time constraints in primary care and appointment delays), we allowed more time in order for them to receive the 4 planned shopping reports over the full trial period.**

11. Line 274: "Intakes of key food groups (%EI) were not statistically significant between intervention and control" - should 'significant' read 'different'; the sentence does not quite scan?

**Answer: We have modified this sentence to clarify this.**

12. Were any particular behavioural components omitted from delivery more frequently than others? It would be interesting to know a little more about the outcome of the fidelity analyses but not essential.

**Answer: We have looked into this and have added a sentence about behavioural components which were missed the most (Line 441-442).**

Discussion

13. "However, the reductions in SFA and LDL-cholesterol in the intervention groups were somewhat greater than those in the control with no formal intervention" - can this claim be modified to remind the reader that such changes (and, later in the Discussion, the change in SFA in purchases) did not reach statistical significance?

**Answer: We have modified this sentence as suggested.**

14. Might the relatively brief duration of the intervention period be a study limitation?

**Answer: We have considered this but do not believe it is likely. We planned the length of the study to detect changes in blood lipids as a result of decreasing saturated fat in the diet. Previous evidence suggest blood lipids can change in as little as 4 weeks which supports our choice of the length of the study. If anything, we might expect the effect to wane over time and hence in this initial testing we kept the follow up short to examine any initial effects of the intervention. Ultimately longer term studies will be required to test the sustainability of any dietary change that may occur.**

15. Given that the trial also assessed the feasibility of the recruitment process, what changes would the team consider to this process given the relatively low response rate of 3% of potentially eligible participants?

**Answer: We appreciate the reviewer comment regarding the feasibility of recruitment. We have included in the discussion a suggestion to include a larger sample of GP practices in order to recruit successfully for a larger trial (line 521-523). In part this low response rate is due to the many demands of the study including the requirement to do most of the household shopping in the participating supermarket (in order for us to access their loyalty card data). However it may also reflect relatively low interest in the population in dietary change. The response rates for our studies testing weight loss interventions is ~10%. We have a ‘chicken and egg’ situation because doctors are unconvinced that interventions in primary care will change diet composition and tend not to encourage patients to make changes. But this perspective may not change until we have evidence – and recruitment to trials is low because of lack of engagement in the issue by practices and patients. Hence at present it may be necessary to recruit across many practices. This solves the feasibility question, but leaves open issues of generalisability of the findings to the whole population with elevated LDL in primary care.**

Abbreviations

16. Might EI and TE be profitably added to the list of abbreviations?

**Answer: We have added those to the list of abbreviations and also disclosed all acronyms in footnotes.**

References

17. Is there an issue with reference 8 (Office of national statistics?) Similarly references 20 and 21. References 33 and 34 seem to be duplicates.

**Answer: We have checked our references to make sure they are complete and not duplicated.**

-----------------------------------------------------------

Reviewer #3: Thank you for the opportunity to review this paper.

This is a small randomized trial (n=113) to 3 groups a Brief Support a Brief support +Shopping feedback and a control group that reports feasibility but no effectiveness. This is an interesting intervention, potentially scalable and a novel idea which needs evaluation, however this is underpowered and can only really establish the feasibility of implementing this strategy and not the effectiveness or otherwise of this strategy. Important partnerships have been established to enable this and likely work done to develop the intervention. Overall this is well written, but I think there could be on balance more emphasis on the feasibility/ implementation aspects and less on the effectiveness.

**Answer: We thank the reviewer for their helpful comments and have tried to address all their concerns. We agree that there is an important component around feasibility of the trial procedures, but the trial was originally planned as an effectiveness trial with appropriate power to detect changes in the primary outcome. But we agree that some of the secondary effectiveness measures lack power overall. As suggested by another reviewer, we have noted the hybrid “effectiveness-feasibility” design of this trial where relevant.**

Methods:

Why did the investigators only target patients with high LDL in the last 5 years? An Absolute risk approach could have been taken, or it could have been justified to target a larger population group as a saturated fat intervention is likely to be relevant to a wider group of patients. Why were patients with CVD excluded? A dietary intervention on-top of there usual treatment would seem reasonable. And the primary outcome was self-reported saturated fat and not LDL cholesterol.

**Answer: Our intention was to recruit people at risk of CVD as a virtue of having raised LDL cholesterol in the previous 5 years since reducing SFA specifically lowers LDL rather than overall CVD risk.**

**We agree that saturated fat reduction would be beneficial at the population level but in this feasibility testing of a new intervention we chose to focus on a group at higher risk, who may be more motivated to change and where there is a greater likelihood of observing benefits.**

**We excluded only patients with serious pre-exciting or recent CVD conditions (e.g. stroke in the last 3 months) as noted in our exclusion criteria but did not exclude people with mild CVD conditions neither those on statins or cholesterol-lowering medication except those planning changes in their treatment during the conduct of the study.**

**We chose the primary outcome to be saturated fat as this is the main target in most dietary interventions that aim to prevent cardiovascular disease. Saturated fat is an important mediator of the association between diet and LDL cholesterol and this trial was a proof of concept that these interventions can help reduce SFA in the diet as a first step to improve LDL cholesterol and the risk of CVD. A subsequent study would need to have a larger sample and longer follow up to prove a reduction in LDL cholesterol and/or CVD risk.**

Why was a 1:3:3 ratio used?

**Answer: We apologise for the lack of clarity regarding the allocation ratio used in this study and this was noted by another reviewer. Our primary outcome compared the change in SFA between each intervention and control (lines 308-310). The difference that we wanted to detect was 3% (SD 3), therefore we needed 16 participants per group. However, we also stated that a secondary comparison (lines 310-311) was to compare the 2 active intervention arms to each other to detect a further difference of 2% (SD 3) between them, for which we needed 48 participants in each of the active arms to account for 10% drop outs, 90% power, alpha=0.05 and multiple comparisons. This was the most efficient way of allowing the necessary power for each of the proposed comparisons.**

Research staff not being blinded to allocation could have been an issue, but the primary outcome was assessed through an electronic questionnaire without involving the study team.

It would be good to describe here or in discussion about the development of the partnership with the UK grocery store in more detail and the design of the shopping report, who was involved in the design and was it tested with consumers prior to the trial. I think this might add more depth to this work.

**Answer: As noted in the acknowledgement section, the partnership established in this project was specifically limited to the provision of grocery shopping data by the retailer to the supermarket. The retailer had no involvement in the initiation of the study, the design of the intervention or analysis of the results. They were provided with a copy of the paper prior to submission only to correct any factual errors relating to their business or to identify any potential breaches of the contract – no changes were requested. Once the data was received, the study team used a bespoke system developed by us to convert grocery shopping data in a month shopping report which was provided to the study participants. This was not tested in other participants before this trial. The details of this process were described in detail in our protocol paper, but we have included some details in the relevant section to clarify this (Line 217-218). We conducted qualitative interviews with participants at the end of the study which will be reported separately and the feedback they provided based on their direct experience of the intervention will be used to refine the algorithm and inform future research in this area.**

Results:

There seems to be some imbalances in some risk factors (e.g. normal weight), this is likely to have been chance given the small sample size of groups, however it would be helpful to see what the distribution of absolute cardiovascular risk was across participants.

**Answer: As noted here and by another reviewer, there was a small imbalance between the trial arms in the average BMI and BMI categories. This is something that we didn’t anticipate so we did not plan to adjust our analyses for BMI. However, we have explored adjustment for BMI in the model and have seen negligible changes to the estimates for the analysis of the main outcome which don’t change the interpretation of the reported results (see supplemental table S7).**

**All the other baseline measures of CVD risk, including blood lipids and blood pressure, can be found in supplementary tables.**

Discussion:

This is generally well discussed. There is some repetition and could probably be trimmed if word count/ length an issue. The focus of conclusions is effectiveness but probably the focus should be feasibility and implementation evaluation. It would be good to emphasize and discuss the learnings from the intervention implementation.

**Answer: We have revised our conclusion and tried to highlight the potential implementation of this intervention given the good evidence of feasibility provided by our study. We will leave it to the editor to decide if the length of the manuscript is an issue.**

The issue of not all the behavioural components being delivered is probably a challenge of implementation and it may be good to discuss how this may be addressed in the future, is it just the training? Or is the HCP step needed? Or are there other ways of delivering this? Or does the content of delivery matter so much as the contact with the HCP?

**Answer: We appreciate the reviewer comment and agree that a potential challenge is the fidelity of the intervention delivery. Although we recognise that the training provided to the HCPs could have been better (e.g. longer training session, refresher sessions, etc), the content provided comes from publicly available leaflets which participants can easily access if they wish without the need to involve the HCP. But participants generally appreciated the contact with the HCP as we observed in qualitative interviews. We are currently preparing a separate qualitative paper to explore these issues.**

In addition:

I would usually think good to include the inclusion/ exclusion criteria and consort diagram in main paper as opposed to appendix.

**Answer: We have included the Consort diagram and inclusion/exclusion criteria in the main manuscript as requested by the editor.**

I think a figure to show an example of the shopping report would also be helpful to visualize what this comprised.

**Answer: We have included an example of the shopping report in the manuscript.**

I think having a consort diagram and figure to show the shopping report would be more helpful than existing figures. E.g. perhaps Figure 1 could be put in appendix (this repeats data in text/tables doesn't it), figure 2 could be kept and figure 3 could be put in appendix.

**Answer: We have kept figure 1 because this is the primary outcome analysis and the actual data only comes as supplemental tables, but have re-ordered the other figures to include the Consort diagram and example of shopping report.**
