# Supplementary material for: Evaluation of an intervention to provide brief support and personalized feedback on food shopping to reduce saturated fat intake (PC-SHOP): A randomized controlled trial
Source: PLoS Med. 2020 Nov 5;17(11):e1003385. doi: 10.1371/journal.pmed.1003385 (PMC7643942; doi:10.1371/journal.pmed.1003385)
Supplement: Attachment — Submitted filename: Second review queries_PCSHOP 24 08 2020.docx [file pmed.1003385.s007.docx]

"Evaluation of an intervention to provide brief support and personalised feedback on food shopping to reduce saturated fat intake (PC-SHOP): a randomised controlled trial"

PMEDICINE-D-19-03921R1

------------------------------------------------------------

Requests from Editors:

Abstract

GP – perhaps not commonly used outside the UK so I would recommend introducing on first view

Please provide brief participant demographics-include age range, sex etc in the abstract

Please provide p-values as needed. Al p-values should be exact numbers, unless p<0.001

**Answer: We have made the changes requested**

Data statement: We understand the data restrictions due to the ethics and contractual obligations. However, please note we do not permit authors to act as gatekeepers to the primary underlying data. Please provide an institutional or ethics committee contact instead. Regarding the Brand View dataset, please provide details of the dataset and contact details of someone at BrandView who can be approached by interested parties to gain access to the data.

**Answer: We have provided more details of the shopping data and Brand View data as follows:**

*“Because of contractual restrictions to the use of the grocery shopping data it is not possible to make this data openly available outside this project but any queries can be directed to the University Research Services (carly.banner@admin.ox.ac.uk) and the Clinical Trials & Research Governance and the Health Research Authority that provided ethical approval (ctrg@admin.ox.ac.uk; nrescommittee.southcentral-oxfordc@nhs.net) Grocery shopping data was supplemented with a dataset containing the nutritional composition of foods provided by a commercial organisation (Brand View). Further use of this data must be negotiated with the data owners Brand View (support@brandview.com)”.*

Author summary

Please add bullet points

Nurse or healthcare professional seems a bit redundant. Could you perhaps just say healthcare professional?

I suggest rephrasing “we followed them for approximately 3 months” as this could mean any number of things

**Answer: We have revised our summary as suggested**

Methods

All references to questionnaires used in the study should be accompanied by a citation to the reference, either as published or as provided in SI files

Line 277- suggest revising “results were averaged…”

**Answer: Both the Oxford WebQ dietary questionnaire and the non-validated qualitative questionnaire have been accompanied by a reference to the published paper (WebQ) or supplementary files (qualitative questionnaire). We have reworded line 277**

CONSORT flowchart-please specify reasons for ineligibility

**Answer: We have included the reasons for ineligibility in our Consort flowchart**

Results

Line 375 and line 397 onwards- please provide all p-values to accompany 95% CI

In all instances where you mention “significance” or “non-significance” please provide 95% CI and p values.

**Answer: We have revised our results section to make sure we have provided p values where appropriate (as justified in our protocol and statistical plan). Please note that we have only provided p values where we have done testing between each intervention group vs control for the main study outcomes. But we have also reported changes from baseline to follow up in each study arm, for which we have not provided p values given that there were only shown in a descriptive way. For all the other non-significant tests performed in other secondary outcomes (which have not been included in the text to avoid using too much text given the large amount of outcomes reported), all p values can be found in the supplementary tables.**

I suggest toning this down “Participants in the control group appeared to have been prompted to reduce their SFA upon receiving their blood test results and the notice of a future check three months later, which reduced the chance of detecting a difference between groups”

**Answer: We have rephrased this as follows:**

*“It is possible that participants in the control group could have been prompted to reduce their SFA upon receiving their blood test”*

CONSORT checklist- please use paragraphs and sections instead of page numbers as these are likely to change

**Answer: We have modified our Consort checklist as suggested**

Overall- please avoid the use of the phrase “somewhat greater” and use significant or non-significant instead.

**Answer: We have rephrased this as follows:**

*“However, the reductions in SFA and LDL-cholesterol in the intervention groups were not significant but larger than those in the control with no formal intervention.”*

Comments from Reviewers:

Reviewer #1: Thanks for the revisions to the manuscript and the details given in the response to my comments (and the other reviewers). The comments and the revisions to the manuscripts have resolved all my original queries.

One comment that may be covered during further editorial process was that the figures have some issues - in my PDF some of the axis label text is obscured by background stippling and the text isn't rendered properly making it difficult to read. This could be something induced by the MS submission system.

**Answer: We have provided separate files for each figure which should have the adequate resolution**

For Figure 3 I'd recommend representing the % change as a dot or square rather than a bar.

**Answer: Figures 3 and 4 represent the change from baseline to follow up in each study arm and we believe that using bars is appropriate for these kinds of figures. However we can change them if the editor considers it necessary.**

Reviewer #2: I'd like to thank the authors very much for their response to my comments and those of the editor and other reviewers - the manuscript is much clearer. The response to comment number 15 in my review regarding engagement from colleagues in primary care in the recruitment process is particularly interesting and illuminating, and very much highlights the need for this sort of study!

I have just one other suggestion for the revised manuscript.

Box describing intervention: Could the description of the shopping report be clarified further please? It currently appears as if the 'report' in point 1 is separate from the information listed in point 2, whereas my understanding is that the report consisted of the information under point 2. Apologies if I have misunderstood. In addition, also in this box, is there any possibility that the number of reports could be specified alongside the statement that they were provided monthly from baseline onwards?

**Answer: We have modified this box as suggested**
